# Systems biology of the IMIDIA biobank from organ donors and pancreatectomised patients defines a novel transcriptomic signature of islets from individuals with type 2 diabetes

**DOI:** 10.1007/s00125-017-4500-3

**Published:** 2017-11-28

**Authors:** Michele Solimena, Anke M. Schulte, Lorella Marselli, Florian Ehehalt, Daniela Richter, Manuela Kleeberg, Hassan Mziaut, Klaus-Peter Knoch, Julia Parnis, Marco Bugliani, Afshan Siddiq, Anne Jörns, Frédéric Burdet, Robin Liechti, Mara Suleiman, Daniel Margerie, Farooq Syed, Marius Distler, Robert Grützmann, Enrico Petretto, Aida Moreno-Moral, Carolin Wegbrod, Anke Sönmez, Katja Pfriem, Anne Friedrich, Jörn Meinel, Claes B. Wollheim, Gustavo B. Baretton, Raphael Scharfmann, Everson Nogoceke, Ezio Bonifacio, Dorothée Sturm, Birgit Meyer-Puttlitz, Ugo Boggi, Hans-Detlev Saeger, Franco Filipponi, Mathias Lesche, Paolo Meda, Andreas Dahl, Leonore Wigger, Ioannis Xenarios, Mario Falchi, Bernard Thorens, Jürgen Weitz, Krister Bokvist, Sigurd Lenzen, Guy A. Rutter, Philippe Froguel, Manon von Bülow, Mark Ibberson, Piero Marchetti

**Affiliations:** 1 Paul Langerhans Institute Dresden (PLID) of the Helmholtz Center Munich at University Hospital Carl Gustav Carus and Faculty of Medicine, TU Dresden, Fetscherstrasse 74, 01307 Dresden, Germany; 2 German Center for Diabetes Research (DZD), Munich Neuherberg, Germany; 3 Max Planck Institute of Molecular Cell Biology and Genetics (MPI-CBG), 01307 Dresden, Germany; 4 Sanofi-Aventis Deutschland GmbH, Diabetes Research, Industriepark Höchst, Building H821, 65926 Frankfurt am Main, Germany; 5 Department of Clinical and Experimental Medicine, Cisanello University Hospital, University of Pisa, Via Paradisa 2, 56126 Pisa, Italy; 6 Department of Visceral-Thoracic-Vascular Surgery, University Hospital Carl Gustav Carus and Faculty of Medicine, TU Dresden, Dresden, Germany; 7 Section of Cell Biology and Functional Genomics, Division of Diabetes, Endocrinology and Metabolism, Imperial Centre for Translational and Experimental Medicine, Imperial College London, London, UK; 8 Queen Mary University of London, Dawson Hall, London, UK; 9 Department of Genomics of Common Disease, School of Public Health, Imperial College London, Hammersmith Hospital, London, UK; 10 Institute of Clinical Biochemistry, Hannover Medical School, Hannover, Germany; 11 Vital-IT Group, SIB Swiss Institute of Bioinformatics, Quartier Sorge, bâtiment Génopode, 1015 Lausanne, Switzerland; 12 Department of Surgery, University Hospital of Erlangen, Erlangen, Germany; 13 Medical Research Council (MRC), Institute of Medical Sciences, Imperial College London, London, UK; 14 Duke-NUS Medical School, Singapore, Republic of Singapore; 15 Department of Pathology, University Hospital Carl Gustav Carus and Faculty of Medicine, TU Dresden, Dresden, Germany; 16 Department of Cell Physiology and Metabolism, Geneva University Medical Center, Geneva, Switzerland; 17 INSERM, U1016, Institut Cochin, Faculté de Médecine, Université Paris Descartes, Sorbonne Paris Cité, Paris, France; 18 F. Hoffmann-La Roche Ltd, Roche Innovation Center Basel, Basel, Switzerland; 19 Center for Regenerative Therapies Dresden (CRTD), TU Dresden, Dresden, Germany; 20 Biotechnology Center, TU Dresden, Dresden, Germany; 21 Centre for Integrative Genomics, University of Lausanne, Lausanne, Switzerland; 22 Lilly Research Laboratories, Eli Lilly, Indianapolis, IN USA; 23 CNRS-UMR8199, Lille Pasteur Institute, Lille, France; 24 Lille University Hospital, Lille, France; 25 European Genomic Institute for Diabetes (EGID), Lille, France

**Keywords:** Beta cell, Biobank, Diabetes, Gene expression, Insulin secretion, Islet, Laser capture microdissection, Organ donor, Pancreatectomy, Systems biology

## Abstract

**Aims/hypothesis:**

Pancreatic islet beta cell failure causes type 2 diabetes in humans. To identify transcriptomic changes in type 2 diabetic islets, the Innovative Medicines Initiative for Diabetes: Improving beta-cell function and identification of diagnostic biomarkers for treatment monitoring in Diabetes (IMIDIA) consortium (www.imidia.org) established a comprehensive, unique multicentre biobank of human islets and pancreas tissues from organ donors and metabolically phenotyped pancreatectomised patients (PPP).

**Methods:**

Affymetrix microarrays were used to assess the islet transcriptome of islets isolated either by enzymatic digestion from 103 organ donors (OD), including 84 non-diabetic and 19 type 2 diabetic individuals, or by laser capture microdissection (LCM) from surgical specimens of 103 PPP, including 32 non-diabetic, 36 with type 2 diabetes, 15 with impaired glucose tolerance (IGT) and 20 with recent-onset diabetes (<1 year), conceivably secondary to the pancreatic disorder leading to surgery (type 3c diabetes). Bioinformatics tools were used to (1) compare the islet transcriptome of type 2 diabetic vs non-diabetic OD and PPP as well as vs IGT and type 3c diabetes within the PPP group; and (2) identify transcription factors driving gene co-expression modules correlated with insulin secretion ex vivo and glucose tolerance in vivo. Selected genes of interest were validated for their expression and function in beta cells.

**Results:**

Comparative transcriptomic analysis identified 19 genes differentially expressed (false discovery rate ≤0.05, fold change ≥1.5) in type 2 diabetic vs non-diabetic islets from OD and PPP. Nine out of these 19 dysregulated genes were not previously reported to be dysregulated in type 2 diabetic islets. Signature genes included *TMEM37*, which inhibited Ca^2+^-influx and insulin secretion in beta cells, and *ARG2* and *PPP1R1A*, which promoted insulin secretion. Systems biology approaches identified *HNF1A*, *PDX1* and *REST* as drivers of gene co-expression modules correlated with impaired insulin secretion or glucose tolerance, and 14 out of 19 differentially expressed type 2 diabetic islet signature genes were enriched in these modules. None of these signature genes was significantly dysregulated in islets of PPP with impaired glucose tolerance or type 3c diabetes.

**Conclusions/interpretation:**

These studies enabled the stringent definition of a novel transcriptomic signature of type 2 diabetic islets, regardless of islet source and isolation procedure. Lack of this signature in islets from PPP with IGT or type 3c diabetes indicates differences possibly due to peculiarities of these hyperglycaemic conditions and/or a role for duration and severity of hyperglycaemia. Alternatively, these transcriptomic changes capture, but may not precede, beta cell failure.

**Electronic supplementary material:**

The online version of this article (10.1007/s00125-017-4500-3) contains peer-reviewed but unedited supplementary material, which is available to authorised users.

## Introduction

The interplay of genetic and environmental factors leads to impaired beta cell function and viability, which are the ultimate and possibly primary causes of type 2 diabetes [1–3]. Studies of post-mortem pancreases from non-diabetic and type 2 diabetic individuals [4, 5] as well as acute diabetes reversal following bariatric surgery [6], pancreatectomy [7] or severe nutrient restriction [8] have challenged the notion that beta cell death is the major cause of type 2 diabetes. Instead, insufficient insulin secretion in type 2 diabetes has been attributed to beta cell dysfunction due to various mechanisms, including de-differentiation [9, 10].

Early studies of islets from non-diabetic (≤7) and type 2 diabetic (≤6) organ donors reported the downregulation (*p* < 0.01) in type 2 diabetic islets of *HNF4A* and *ARNT* [11], known to regulate exocytosis, mitochondrial activity [12, 13] and islet structure and function [14]. Groop and collaborators correlated the transcriptome of islets from 54 non-diabetic and nine type 2 diabetic organ donors with ex vivo insulin secretion and clinical features, including HbA_1c_ [15]. The same group also compared the islet transcriptome of 51 non-diabetic individuals, 12 type 2 diabetic individuals and 15 people with an HbA_1c_ of 6.0–6.5% (42–48 mmol/mol) [16]. These studies found several genes to be differentially expressed in type 2 diabetic islets, including some related to insulin secretion and/or HbA_1c_ [15, 16], beta cell apoptosis [17] and beta cell proliferation [18, 19]. Furthermore, in type 2 diabetic islets, they described upregulation of *SFRP4*, which might link islet inflammation to beta cell dysfunction [20].

Two recent studies analysed the transcriptomes of single islet cells from 12 or six non-diabetic individuals, and six or four type 2 diabetic organ donors, respectively [21, 22], and identified 48 [21] or 75 [22] differentially expressed transcripts in type 2 diabetic islet beta cells. Only one gene (*FXYD2*) was regulated in both studies, but in the opposite direction. Thus, a consensus on transcriptomic alterations in type 2 diabetic beta cells is still lacking. In fact, seven transcripts reported as being regulated (non-overlapping between the two studies) had previously been found to be dysregulated in type 2 diabetic vs non-diabetic beta cells yielded by laser capture microdissection (LCM) from ten non-diabetic and ten type 2 diabetic organ donors [23].

While all these studies [11–23] provided insights into the molecular changes of islets and beta cells in type 2 diabetes, diversity in the number of cases, islet and cell handling, platforms and analytic procedures could account for their different outcomes. Furthermore, transcriptomic data were generally obtained from enzymatically isolated islets; in just one instance, beta cells were retrieved by LCM [23]. To identify robust gene expression changes in type 2 diabetic islets independent of recruitment centre, source (organ donor vs phenotyped pancreatectomised patients [PPP]) and isolation procedure (enzymatic digestion vs LCM), the Innovative Medicines Initiative for Diabetes: Improving beta-cell function and identification of diagnostic biomarkers for treatment monitoring in Diabetes (IMIDIA) consortium (www.imidia.org) established a large multicentre biobank of human islets from organ donors and PPP. The aim of this innovative approach was to identify consistent transcriptomic changes in type 2 diabetic islets and to evaluate their presence in islets from PPP with glucose intolerance or type 3c diabetes.

## Methods

For detailed Methods, please refer to the electronic supplementary material (ESM).

### Islet procurement, insulin secretion and RNA extraction

Pancreases unsuitable for transplantation were obtained in Pisa from 161 non-diabetic and 39 type 2 diabetic heart-beating organ donors (OD) with the approval of the local ethics committees. Type 2 diabetes was diagnosed based on clinical history, treatment with glucose-lowering drugs, and lack of anti-GAD65 autoantibodies. Islets were successfully isolated from the pancreases of 153 non-diabetic and 34 type 2 diabetic OD by enzymatic digestion and density gradient purification, and their insulin secretion was assessed using an immunoradiometric assay, as previously described [5, 24]. RNA was purified 2–3 days after islet isolation (see ESM Methods for further details). Forty-three additional human islet preparations, all from non-diabetic OD, were acquired by Eli Lilly from Prodo Laboratories (Irvine, CA, USA). Please see ESM Methods for further details.

Pancreatic surgical specimens were obtained in Dresden from 201 PPP following patients’ informed consent and approval by the local ethics committee. Islets specimens were retrieved by LCM from snap-frozen surgical specimen of 117 PPP (37 non-diabetic, 41 type 2 diabetic, 16 with impaired glucose tolerance (IGT) and 23 type 3c diabetic individuals) and their RNA was purified as previously described [25]. Please see ESM Methods for further details.

Human islet beta and alpha cell-enriched fractions were prepared from islets isolated in Pisa from OD, as previously described [26] (see ESM Methods, for more details).

### RNA sequencing of OD islets exposed ex vivo to hyperglycaemia

Three independent islet preparations from non-diabetic OD (age: 80 ± 4 years, sex: 1 female/2 male, BMI: 22.7 ± 0.6 kg/m^2^) were used to assess islet gene expression after exposure to 22.2 mmol/l glucose vs 5.5 mmol/l glucose (see ESM Methods for further details).

### Microarrays

RNA was extracted from the islet samples, processed and subjected to transcriptomic profiling as described in the ESM Methods (‘Extraction of RNA from islets isolated enzymatically or by LCM from PPP surgical specimens’. and ‘RNA quality assessment, processing and transcriptomic profiling’). Microarray findings were validated by transfection of cDNA vectors in Chinese hamster ovary [CHO] cells (*ARG2*, *PPP1R1A* and *TMEM37*) and INS-1 832/13 cells (*Tmem37-*V5) or silencing RNAs in INS-1 832/13 (*Arg2*, *Ppp1r1a* and *Tmem37*) and EndoC-βH1 cells (*ARG2*, *PPP1R1A* and *TMEM37*), Ca^2+^ imaging, reverse transcription quantitative PCR (RT-qPCR) (*ACTB*, *ANKRD23*, *ANKRD39*, *ARG2*, *ASCL2*, *CAPN13*, *CD44*, *CHL1*, *FAM102B*, *FBXO32*, *FFAR4*, *G6PC2*, *HHATL*, *HNF1A*, *KCNH8*, *NSG1*, *PCDH20*, *PDX1*, *PPP1R1A*, *SCTR*, *SLC2A2*, *TMEM37*, *UNC5D*), in situ RT-PCR (*ARG2*, *ASCL2*, *CHL1*-iso1, *CHL1*-iso2, *CHL1*-iso3, *FAM102B*, *FFAR4*, *HHATL*, *KCNH8*, *PPP1R1A*, *SCTR*, *SLC2A2*, *TMEM37* and *UNC5D*), western blotting (V5 epitope tag, γ-tubulin, pancreatic and duodenal homeobox 1 [PDX1] and HNF1 homeobox A [HNF1A]), immunomicroscopy (insulin, protein phosphatase 1 regulatory inhibitor subunit 1A [PPP1R1A], transmembrane protein 37 [TMEM37], glucagon and arginase 2 [ARG2]) and chromatin immunoprecipitation (PDX1 and HNF1A). See the ESM Methods for further details. Primers are listed in ESM Tables 1–4.

### Data analysis

#### Processing and analysis of RNA sequencing data from islets exposed ex vivo to hyperglycaemia

Single-end reads (75 bp) were aligned to human genomic sequence (hg19 assembly) using TopHat and Bowtie2 (version 2.0.11 and version 2.2.1, respectively; http://ccb.jhu.edu/software.shtml) and using Samtools (version 0.1.19; http://samtools.sourceforge.net/) for sorting of alignment files. Read counts per gene were then generated using HTSeq (htseq-count version 0.5.4p3; https://htseq.readthedocs.io/) and Ensembl genome annotation 75 (http://feb2014.archive.ensembl.org/index.html). Differentially expressed genes were detected using either DESeq2 (version 1.15.51) or Limma (version 3.30.12) from the Bioconductor software package (https://bioconductor.org/) in the R statistical programming environment (https://www.r-project.org/). For DESeq2, single-end reads (75 bp) were aligned to human genomic sequence (hg38 assembly) using GSNAP (version 2017-03-17; http://research-pub.gene.com/gmap/), and Ensembl annotation 87 was used to detect reads spanning splice sites. The uniquely aligned reads were counted with featureCounts (version 1.5.2, Bioconductor) and the same Ensembl annotation. The raw counts were normalised based on the library size and testing for differential gene expression between the two conditions, samples treated with glucose vs control, was performed with the DESeq2 R package. For Limma, the raw count data were first filtered for an average of at least five reads in all the samples, normalised to library size using the weighted trimmed mean of M-values (TMM) method in edgeR (version 3.16.5, Bioconductor), and then transformed to log_2_-cpm (counts per million reads) using the voom function in R. Empirical Bayes moderated t statistics and corresponding *p* values were then computed comparing the samples treated with glucose to controls using the Limma package in R. The *p* values were adjusted for multiple comparisons using the Benjamini–Hochberg procedure in R.

#### Normalisation and statistical analysis of microarray data

Transcriptomics data were normalised by Robust Multi-array Average (RMA) in Array Studio software (Omicsoft, Cary, NC, USA). Batch correction of microarray data was performed using the Bioconductor package ComBat in R. Elimination of technical outlier samples was performed at two steps of the transcriptomics analysis. The criterion for expression was an intensity value of >75% for ≥25% of the samples in that group. Subsequently, islet samples from organ donors with no previous history of diabetes but with blood fructosamine >285 μmol/l or glucose >11.1 mmol/l were excluded from the analysis. Islet samples from non-diabetic/type 2 diabetic OD with insulin levels <1 SD from the within-group mean were also excluded. For comparisons between type 2 diabetic and non-diabetic OD islets, significant differences were defined as a change in expression of ≥1.5 after correction for multiple hypothesis testing using the Benjamini–Hochberg method (*p* ≤ 0.05). Principal component analysis was performed using the prcomp in R. The analysis of all islet sample types and of a single sample type (islets from OD or PPP) was based on the intensities of the probe sets after batch correction. Contamination of islet samples with exocrine pancreatic tissue was determined using selected markers of exocrine and ductal cells, as indicated in ESM Table 5.

#### Bioinformatics

The bioinformatics analyses performed with Ingenuity Pathway Analysis software (Qiagen, Hilden, Germany) comprised gene ontology over-representation analysis of differentially expressed genes; determination of enriched genes involved in insulin secretion; and pathway analysis of differentially expressed genes. Other bioinformatics analysis comprised prediction of binding sites for HNF1A and PDX1 in type 2 diabetic islet signature genes; identification of gene co-expression modules from OD islet and PPP-LCM samples; generation of a trait module network; identification of module hub genes and measurement of the overlap with signature genes; and generation and merging of sequence-based and library-based transcription factor networks. The signature genes were *ANKRD23*, *ANKRD39*, *ARG2*, *ASCL2*, *CAPN13*, *CD44*, *CHL1*, *FAM102B*, *FBXO32*, *FFAR4*, *G6PC2*, *HHATL*, *KCNH8*, *NSG1*, *PCDH20*, *PPP1R1A*, *SCTR*, *SLC2A2*, *TMEM37* and *UNC5D*. See ESM Methods (‘Data analysis’ section) for further details.

## Results

### The IMIDIA cohorts

**Fig. 1:**
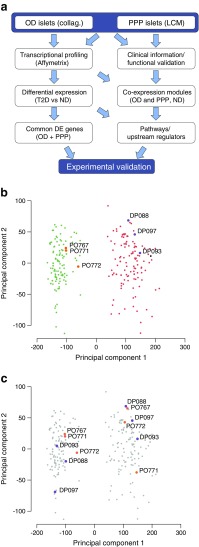
Transcriptional profiling of islet samples using complementary isolation techniques. (**a**) Overview of our approach. Islets from OD and PPP were analysed by Affymetrix profiling to identify differentially expressed (DE) genes. These data were combined with clinical and functional data to identify gene co-expression modules correlated with T2D-related traits. ‘Collag.’ refers to enzymatic digestion. ‘Common DE genes’ refers to genes differentially regulated in T2D vs ND in both OD and PPP islets. ‘Pathways/Upstream regulators’ refers to pathways downstream of and regulatory genes upstream of gene co-expression modules. (**b**, **c**). Principal component analysis of OD and PPP islets isolated enzymatically or by LCM using all transcribed genes. Principal component 1 (PC1) accounts for 49% of the total variance; PC2 accounts for 4%. (**b**) LCM islet samples (green, PPP; orange, OD) cluster separately from enzymatically isolated islet samples (orange, OD; grey, PPP) regardless of patient origin. (**c**) Duplicate OD (orange) and PPP (purple) samples isolated either enzymatically or by LCM are highlighted, confirming that clustering is according to the isolation method. DP, code for Dresden pancreatectomised patient samples; ND, non-diabetic; PO, code for Pisa OD samples; T2D, type 2 diabetic

We collected pancreatic specimens from two cohorts (Fig. 1a and ESM Table 6). One cohort consisted of 243 OD, including 204 non-diabetic and 39 with type 2 diabetes. As expected, blood fructosamine, a biomarker for glucose levels in the days preceding organ donation, was greater in type 2 diabetic (222 ± 72 μmol/l, *n* = 11) than in non-diabetic OD (180 ± 45 μmol/l, *n* = 46, *p* = 0.018). The second cohort included 201 PPP who underwent pancreatectomy for pancreatic diseases. Among PPP, 70 were non-diabetic, 54 had type 2 diabetes, 30 had IGT, and 46 had diabetes that was likely due to the pancreatic disorder leading to surgery (type 3c diabetes) [27] (see ESM Methods). A diagnosis of type 3c diabetes was made if diabetes was first detected <1 year before the symptoms, which led to surgery. Histopathology of the resected tissue did not reveal insulitis in any PPP. Assessment of insulin secretion by OD islets showed reduced release from type 2 diabetic beta cells in response to glucose or glibenclamide (known as glyburide in the USA and Canada), but not to arginine (ESM Fig. 1), complementing previous findings [24].

Islets were isolated from 141 OD (115 non-diabetic and 26 type 2 diabetic), and 117 PPP (37 non-diabetic, 16 IGT, 41 type 2 diabetic, 23 type 3 diabetic) by enzymatic digestion or LCM. Following filter selection (see ESM Methods) the transcriptomes of islets from 103 OD (84 non-diabetic and 19 type 2 diabetic), and 103 PPP (32 non-diabetic, 36 type 2 diabetic, 15 IGT, 20 type 3c diabetic) were profiled (Table 1). Hence, in total we profiled the islet transcriptomes of 116 non-diabetic OD (84) and PPP (32) and of 55 type 2 diabetic OD (19) and PPP (36). Between the OD and PPP groups, the non-diabetic and type 2 diabetic patients were similar in terms of their age, BMI, and mean duration of diabetes (Table 1). Among non-diabetic and type 2 diabetic PPP, the prevalence of chronic pancreatitis and of benign/malignant tumours was also similar (Table 1).

**Table 1:** Clinical characteristics of the OD and PPP cohorts included in this study

	OD cohort (*n*=103)		PPP cohort (n=103)			
Variable	ND, *n*=84 (81.6%)	T2D, *n*=19 (18.4%)	ND, *n*=32 (31.0%)	T2D, *n*=36 (35.0%)	IGT, *n*=15 (14.6%)	T3cD, *n*=20 (19.4%)
Sex (female/male)	46/38	6/13	16/16	13/23	6/9	6/14
Age (years)	60±16	72±7**	60±14	66±12	63±13	66±9
BMI (kg/m²)	25.8±4.2 (*n*=83)	26.5±3.6	24.9±3.4	25.8±5.0	25.7±3.5	26.0±3.9
Diabetes duration (years)	–	9.9±7.4 (*n*=16)	–	10.6±8.6	–	0.02±0.1
Blood glucose in ICU (mmol/l)	8.0±1.8 (*n*=59)	11.7±4.3**	–	–	–	–
Fasting glucose (mmol/l)	–	–	5.3±0.6 (*n*=27)	8.0±2.7*** (*n*=30)	5.3±0.4	6.7±1.7**
HbA_1c_ (mmol/mol)	–	–	38±6.6 (*n*=31)	58±15.3*** (*n*=34)	40±3.3	46±9.8**
HbA_1c_ (%)	–	–	5.6±0.6 (*n*=31)	7.5±1.4*** (*n*=34)	5.8±0.3	6.4±0.9**
Blood glucose at 2 h in the OGTT (mmol/l)	–	–	6.1±1.3 (*n*=27)	–	9.2±0.8***	12.0±1.4*** (*n*=12)
Histopathology	–	–				
Chronic pancreatitis	–	–	6 (18.7%)	7 (19.4%)	2 (13.3%)	4 (20.0%)
Benign tumour	–	–	8 (25.0%)	6 (16.7%)	5 (33.3%)	1 (5.0%)
Malign tumour	–	–	18 (56.3%)	23 (63.9%)	8 (53.3%)	15 (75.0%)

Except for sex, the values are means ± SD

T2D patients were treated as follows: 16 OD and 13 PPP with oral glucose-lowering agents, 1 OD and 17 PPP with insulin, 1 OD and 4 PPP with oral glucose-lowering agents and insulin, 1 PPP with oral glucose-lowering agents, insulin and liraglutide, and 1 OD and 1 PPP with diet only. The tumour was located in the pancreas head in 13/15 T3cD PPP with an adenocarcinoma, and in the pancreas tail in the other patients. One PPP had a serous microcystic adenoma in the pancreas body

ICU, intense care unit; ND, non-diabetic individual; T2D, type 2 diabetic individual; T3cD, type 3c diabetic individual

**p < 0.01 and ***p < 0.001) vs ND, two-tailed *t* test

### Differentially expressed genes in type 2 diabetic islets

**Fig. 2:**
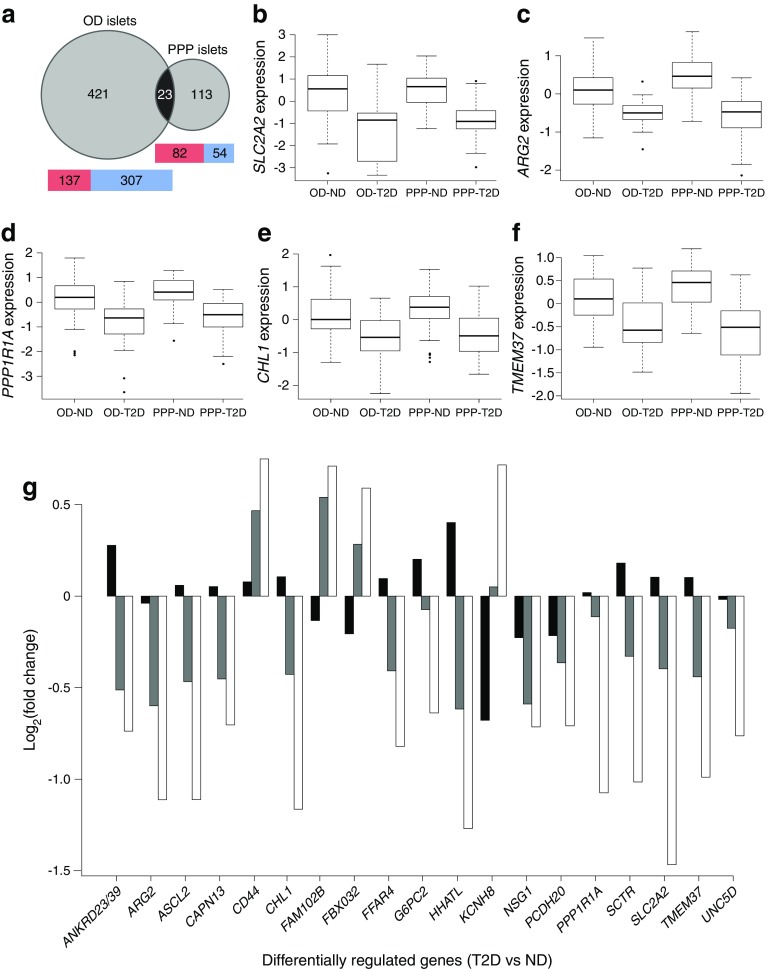
Transcriptomic analyses revealed a common gene signature in type 2 diabetic islets of OD and PPP. (**a**) Venn diagram showing the number of differentially expressed (DE) genes in T2D OD and PPP islets and in ND OD and PPP islets. The numbers include genes mapping to more than one probe set. Twenty-three differentially expressed genes overlapped between the two datasets (*p* = 9.1 × 10^−12^; hypergeometric test assuming a background of 15,165 expressed genes). Heatmaps indicate the number of upregulated (red) and downregulated (blue) genes in OD and PPP islets. (**b**–**f**). Box plots for the expression changes of five of the 19 differentially expressed genes that were dysregulated in the same direction in T2D compared to ND in OD and PPP islets. Circles represent outliers. (**g**) Fold changes in the expression of the 19 commonly dysregulated genes in T2D in islets from IGT PPP (black bars), T3cD PPP (grey bars) and T2D PPP (white bars) vs expression in ND PPP islets. See ESM Tables 7 and 8 for more details. ND, non-diabetic; T2D, type 2 diabetic; T3cD, type 3c diabetic

In total, 4438 out of 29,529 probe sets, corresponding to 2976 unique genes, were differentially expressed in type 2 diabetic vs non-diabetic OD islets (false discovery rate [FDR] ≤0.05). Overall, the expression of 608 probe sets, corresponding to 444 gene annotations (of which 421 were regulated only in OD and not in PPP; ESM Table 7) changed ≥1.5 fold (Fig. 2a and GEO, accession number: GSE76896). Among the top 20 regulated genes, 18 were downregulated, and two were upregulated (ESM Table 8). A total of 1439 of 29,612 probe sets were differentially expressed in type 2 diabetic compared with non-diabetic PPP islets (FDR ≤0.05), corresponding to 1039 unique genes. Overall, the expression of 208 probe sets, corresponding to 136 gene annotations (of which 113 were regulated only in PPP and not in OD; ESM Table 7), changed ≥1.5 fold (Fig. 2a and GEO, accession number: GSE76896). Among the top 20 expressed genes, 12 were downregulated and eight upregulated (ESM Table 8). In type 2 diabetic OD islets, 69% (307/444) of the differentially regulated genes were downregulated, whereas 62% (84/136) of the differentially regulated genes were upregulated in type 2 diabetic PPP islets (Fig. 2a). Exocrine and ductal pancreatic markers were comparably low in OD and PPP islets (see ESM Table 5). Furthermore, islets isolated enzymatically from OD and PPP clustered together by principal component analysis (PCA) and separately from the cluster of islets isolated by LCM from the same OD and PPP (Fig. 1b, c), suggesting the influence of the isolation procedure (enzymatic for OD and LCM for PPP) rather than differences between OD and PPP. Comparing differentially expressed genes with pancreatic cancer transcriptomic signatures (see ESM Results) we found no evidence for contamination of PPP samples with cancer cells [28].

### Dysregulated genes common to type 2 diabetic OD and PPP islets

To identify the most reproducible transcriptomic changes in type 2 diabetic islets independent from covariates such as islet retrieval procedure, tissue source and/or collecting centre, we focused on genes significantly dysregulated in type 2 diabetic islets in both cohorts. This allowed us to identify 23 genes with an FDR of ≤0.05 and a fold change of ≥1.5, of which 19 were dysregulated in the same direction in both type 2 diabetic OD and PPP islets (Table 2 and Fig. 2a). The reason for the regulation in opposite directions of *DAB1*, *GAP43*, *PDK4* and *RGS16* in type 2 diabetic OD and PPP islets is unclear. Fifteen genes were downregulated, including *SLC2A2*, *ARG2*, *CHL1*, *PPP1R1A*, *TMEM37* (Fig. 2b–f), *G6PC2* and *CAPN13*, while four were upregulated (*KCNH8*, *FAM102B*, *FBXO32* and *CD44*). These 19 genes were correlated with stimulated insulin secretion from OD islets (ESM Fig. 2). Notably, nine of these genes, namely *ANKRD23/39*, *ASCL2*, *HHATL*, *NSG1*, *PCDH20*, *SCTR*, *CD44*, *FAM102B* and *FBXO32* have not been previously reported to be dysregulated in type 2 diabetes.

**Table 2:** Genes showing differential regulation in T2D OD and PPP islets (adjusted *p* ≤ 0.05, absolute fold change ≤1.5)

				Islet OD			LCM islets PPP		
No.	Entrez ID	Symbol	Gene name	Probe ID	Ratio	Adj. *p*	Probe ID	Ratio	Adj. *p*
1	51239/200539	*ANKRD23/39*	Ankyrin repeat domain 23/39	229052_at	0.585	1.14 × 10^−2^	1553366_s_at	0.600	1.80 × 10^−3^
2	384	*ARG2*	Arginase 2	203946_s_at	0.605	3.28 × 10^−4^	203946_s_at	0.463	1.47 × 10^−9^
3	430	*ASCL2*	Achaete-scute complex homologue 2	229215_at	0.540	2.52 × 10^−3^	229215_at	0.463	1.74 × 10^−8^
4	92291	*CAPN13*	Calpain 13	229499_at	0.535	2.52 × 10^−3^	229499_at	0.614	4.66 × 10^−3^
5	10752	*CHL1*	Cell adhesion molecule with homology to L1CAM	204591_at	0.411	1.61 × 10^−3^	204591_at	0.446	1.56 × 10^−5^
6	338557	*FFAR4*	Free fatty acid receptor 4	240856_at	0.604	1.10 × 10^−2^	240856_at	0.566	1.79 × 10^−3^
7	57818	*G6PC2*	Glucose-6-phosphatase, catalytic, 2	221453_at	0.616	3.43 × 10^−2^	221453_at	0.643	2.77 × 10^−2^
8	57467	*HHATL*	Hedgehog acyltransferase-like	223572_at	0.388	1.09 × 10^−4^	223572_at	0.415	4.44 × 10^−4^
9	27065	*NSG1*	Neuron specific gene family member 1	209569_x_at	0.624	2.41 × 10^−2^	209569_x_at	0.610	8.59 × 10^−4^
10	64881	*PCDH20*	Protocadherin 20	232054_at	0.628	2.98 × 10^−2^	232054_at	0.612	4.02 × 10^−2^
11	5502	*PPP1R1A*	Protein phosphatase 1, regulatory subunit 1A	235129_at	0.419	3.07 × 10^−4^	235129_at	0.475	5.54 × 10^−6^
12	6344	*SCTR*	Secretin receptor	1565737_at	0.531	4.68 × 10^−4^	1565737_at	0.495	8.30 × 10^−4^
13	6514	*SLC2A2*	Solute carrier family 2, member 2	206535_at	0.273	1.65 × 10^−4^	206535_at	0.362	1.74 × 10^−8^
14	140738	*TMEM37*	Transmembrane protein 37	1554485_s_at	0.654	3.90 × 10^−3^	227190_at	0.504	3.84 × 10^−9^
15	137970	*UNC5D*	Unc-5 homologue D	231325_at	0.535	1.29 × 10^−2^	231325_at	0.589	1.20 × 10^−2^
16	960	*CD44*	CD44 molecule	217523_at	1.561	2.30 × 10^−2^	1557905_s_at	1.681	1.84 × 10^−2^
17	284611	*FAM102B*	Family with sequence similarity 102, member B	226568_at	1.538	9.05 × 10^−3^	226568_at	1.636	2.15 × 10^−2^
18	114907	*FBXO32*	F-box protein 32	232729_at	1.716	1.51 × 10^−3^	225328_at	1.505	7.66 × 10^−3^
19	131096	*KCNH8*	Potassium voltage-gated channel, subfamily H, member 8	1552742_at	1.529	1.09 × 10^−2^	1552742_at	1.644	1.74 × 10^−2^
20	1600	*DAB1*	Dab, reelin signal transducer, homologue 1 (*Drosophila*)	228329_at	0.616	3.90 × 10^−2^	228329_at	1.551	4.59 × 10^−2^
21	2596	*GAP43*	Growth associated protein 43	204471_at	0.611	1.94 × 10^−2^	204471_at	1.880	2.33 × 10^−2^
22	5166	*PDK4*	Pyruvate dehydrogenase kinase, isozyme 4	1562321_at	0.537	8.37 × 10^−3^	1562321_at	2.120	4.06 × 10^−2^
23	6004	*RGS16*	Regulator of G protein signalling 16	209324_s_at	0.663	2.23 × 10^−2^	209324_s_at	1.687	3.80 × 10^−2^

See ESM Tables 7, 8 and 13 for more details

Genes 1–15 are downregulated in OD and PPP; genes 16–19 are upregulated in OD and PPP; and genes 20–23 are downregulated in OD and upregulated in PPP

Probe ID, probe set ID; Adj. *p*, *p* value adjusted for multiple hypothesis tests using the Benjamini–Hochberg method; T2D, type 2 diabetes

Meta-analysis of a published transcriptomic dataset [20] revealed dysregulation (FDR ≤0.05) of 114 probe sets, corresponding to 94 unique genes. We searched this dataset for the 19 genes dysregulated in both our islet cohorts and found that *CHL1*, *FFAR4* and *SLC2A2* were downregulated with an FDR of ≤0.05 and a fold change of ≥1.5, while *ANKRD23/39*, *ARG2*, *HHATL*, *PPP1R1A* and *UNC5D* were downregulated with an FDR of ≤0.1 in type 2 diabetic islets, thus confirming the significant downregulation of *CHL1*, *FFAR4* and *SLC2A2* in type 2 diabetic OD islets in a different cohort.

### Dysregulated genes in IGT and type 3c diabetic islets

The availability of islets from PPP with IGT or type 3c diabetes (Table 1) allowed us to investigate the expression of the 19 genes commonly dysregulated in type 2 diabetic islets according to the extent of hyperglycaemia (Fig. 2g). The expression of the genes differed significantly only between type 2 diabetic and non-diabetic PPP islets (FDR ≤0.05). Nonetheless, the fold changes between type 3c diabetic and non-diabetic PPP islets were in the same direction, albeit with smaller differences than between type 2 diabetic and non-diabetic PPP islets. The fold changes in IGT vs non-diabetic PPP islets were low (≤1.6); only three of the 19 genes had absolute fold changes >1.2. These diversities between islets from type 2 diabetics (both OD and PPP), IGT (PPP) and type 3c diabetics (PPP) may be due to idiosyncrasies of these conditions and/or different duration and severity of the hyperglycaemia.

### Validation of selected genes

Some (*SLC2A2*, *CHL1*, *PPP1R1A*, *ARG2* and *TMEM37*), but not all, of the 19 differentially expressed genes in type 2 diabetic OD and PPP islets were previously shown to be enriched in beta cells and altered in type 2 diabetes [15]. Among the 19 genes dysregulated in type 2 diabetic islets, *ARG2* and *PPP1R1A* were also differentially expressed in non-diabetic OD islets exposed ex vivo to high glucose (22.2 mmol/l) for 48 h, while *CHL1*, *FBX032* and *SLC2A2* showed a trend towards dysregulation (ESM Table 9). These results, although obtained with relatively few preparations (*n* = 3), suggest that the expression of several of the 19 signature genes changes within a relatively short time span upon islet exposure to a ‘hyperglycaemic’ milieu. Confirmation with more samples will be required to better ascertain the precise regulation of these genes in islets upon glucose treatment.

**Fig. 3:**
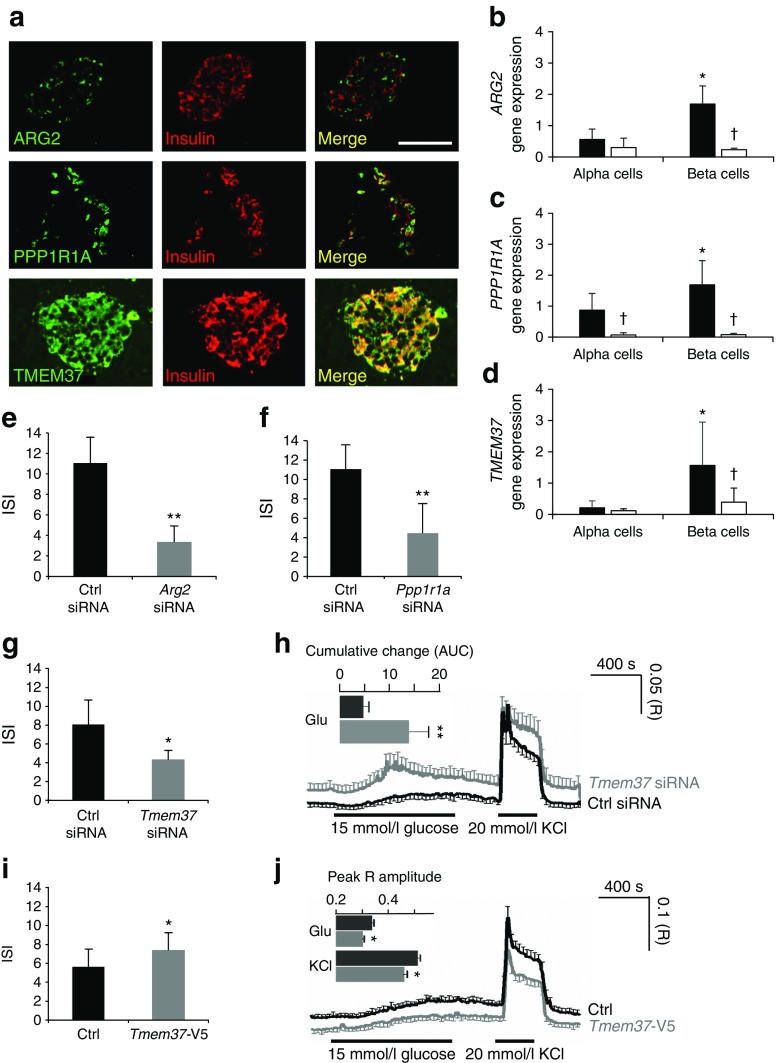
Functional validation of the dysregulated genes *ARG2*, *PPP1R1A* and *TMEM37* in insulin-producing cells. (**a**) Confocal microscopy of human pancreas tissue sections co-immunostained for insulin and ARG2, PPP1R1A or TMEM37. (**b**-**d**) RT-qPCR analysis of *ARG2*, *PPP1R1A* and *TMEM37* expression levels in human islet alpha and beta cell-enriched fractions from ND (*n* = 5, black columns) and T2D (*n* = 4, white columns) OD (*p < 0.05, beta vs alpha cells; ^†^ *p* < 0.05 T2D vs ND beta cells, Student’s *t* test). (**e**-**g**) Insulin stimulation index (ISI) of INS-1 832/13 cells after silencing of *Arg2* (**e**), *Ppp1r1a* (**f**) or *Tmem37* (**g**) expression with small interfering RNA (siRNA) (grey columns) vs cells treated with a control (Ctrl siRNA, black columns) siRNA oligonucleotide (*p < 0.05, **p < 0.01, Student’s *t* test). (**h**) Ca^2+^ concentrations in INS-1 832/13 cells after silencing of *Tmem37* (grey trace) vs cells treated with a control (Ctrl siRNA) siRNA oligonucleotide (black trace). The curves show the mean ± SEM Fura-2 AM ratios for 11 (*n* = 351 siGLO^+^, Ctrl siRNA-treated cells) and 12 (*n* = 480 siGLO^+^, *Tmem37* siRNA-treated cells) coverslips. Changes in glucose and KCl concentrations are indicated. The inset shows the mean ± SEM cumulative Ca^2+^ changes (AUC) in response to glucose (**p < 0.01 vs Ctrl, Mann–Whitney *U* test). (**i**) ISI of INS-1 832/13 cells transfected with *Tmem37-*V5 or the empty pcDNA3.1 vector (Ctrl) (*p < 0.05, Student’s *t* test) (**j**) Ca^2+^ concentrations in eGFP^+^ INS-1 832/13 cells co-transfected with *Tmem37-V5* (grey trace) and eGFP^+^ INS-1 832/13 cells co-transfected with the empty pcDNA3.1 vector (Ctrl) (black trace). The curves show the mean ± SEM Fura Red ratios (R) for ten (*n* = 332 eGFP^+^, *Tmem37-V5* co-transfected cells) and 12 (*n* = 419 eGFP^+^, pcDNA3.1 co-transfected cells) coverslips. The glucose concentration was increased from 3 to 15 mmol/l, and 20 mmol/l KCl was added at the indicated times. The inset shows mean + SEM of the peak R amplitude in response to high glucose and KCl stimulation (*p < 0.05, unpaired two-tailed *t* test). ND, non-diabetic; T2D, type 2 diabetic

Consistent with recent RNA sequencing data of sorted adult beta cells (*n* = 7, non-diabetic cells) [29], in situ PCR on human pancreas sections confirmed islet expression of the 19 type 2 diabetes signature genes (ESM Fig. 3 shows images for three representative genes). As proof of principle, we verified protein expression and localisation of *ARG2*, *PPP1R1A* and *TMEM37* in human pancreas. *ARG2* was detected in a subset of insulin-positive and glucagon-positive cells (Fig. 3a, ESM Fig. 4). Conversely, *PPP1R1A* was co-localised with insulin-positive cells, but its expression was weaker in glucagon-positive cells. *TMEM37* was co-localised with insulin-positive cells and some glucagon-positive cells. Analysis of islet alpha and beta cell-enriched fractions from five non-diabetic and four type 2 diabetic OD by RT-qPCR showed that *ARG2*, *PPP1R1A* and *TMEM37* were enriched in beta cells and downregulated in type 2 diabetes (Fig. 3b–d).

Silencing of either *Arg2* (ESM Fig. 5a) or *Ppp1r1a* (ESM Fig. 5b) in insulinoma INS-1 832/13 cells reduced insulin secretion (Fig. 3e, f), while the opposite was observed by silencing *Tmem37* (Fig. 3g, ESM Fig. 5c), consistent with the latter being an inhibitory subunit of voltage-gated calcium channels [30]. Accordingly, *Tmem37* downregulation increased the proportion of cells with elevated intracellular Ca^2+^ ([Ca^2+^]_i_) concentrations (ESM Fig. 5d, e), and the peak [Ca^2+^] amplitudes (Fig. 3h, ESM Fig. 5f) after exposure to high glucose. Overexpression of *mTmem37-*V5 (ESM Fig. 5g, h) reduced insulin release (Fig. 3i) and [Ca^2+^]_i_ under basal conditions (ESM Fig. 5i) or in response to high glucose or potassium (Fig. 3j). These data suggest that insulin secretion is inhibited by downregulation of *ARG2* and *PPP1R1A* and enhanced by downregulation of *TMEM37*.

### Distinct sets of upstream and downstream pathways are predicted for the OD and PPP cohorts

**Fig. 4:**
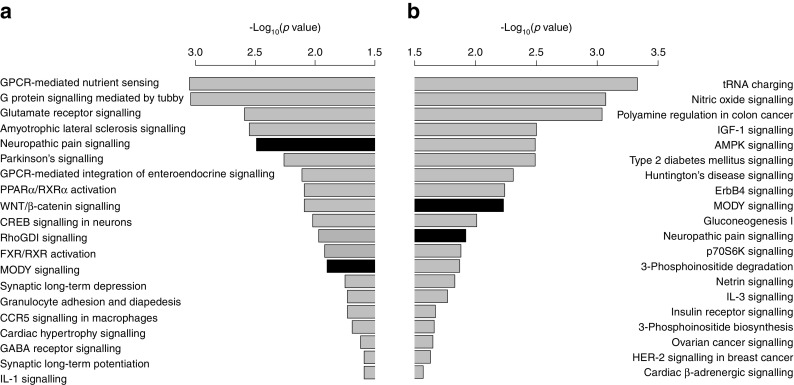
Genes regulated in type 2 diabetic OD and PPP islets are enriched for beta cell function-related pathways. The significance of pathway enrichment is shown as the *–*log_10_(enrichment *p* value) for significantly differentially expressed genes (Limma empirical Bayes adjusted *p* ≤ 0.05, absolute fold change ≤1.5 for OD and ≤1.2 for PPP) in OD (**a**) and PPP (**b**) islets. Black bars represent regulated pathways in common between OD and PPP type 2 diabetic islets. AMPK, 5´ AMP-activated protein kinase; CCR5, C-C motif chemokine receptor 5; CREB, cAMP responsive element binding protein; FXR, farnesoid X receptor; GABA, γ-aminobutyric acid; GPCR, G protein-coupled receptor; HER2, human epidermal growth factor receptor 2; p70S6K, p70 ribosomal protein S6 kinase; PPARα, peroxisome proliferator-activated receptor α; RhoGDI, rho GDP dissociation inhibitor; RXRα, retinoid X receptor α. See ESM Tables 10–12 for more details

Among the enriched pathways related to dysregulated genes identified in type 2 diabetic islets (Fig. 4), several were previously found to influence beta cell function. ‘maturity onset diabetes of young (MODY) signalling’ and ‘neuropathic pain signalling’ were the only two pathways in common among the top 20 identified in each cohort separately. Differentially expressed probe sets separated for upregulation and downregulation were analysed for enriched gene ontologies. Interestingly, similar biological processes were enriched for downregulated probe sets in OD and PPP islets (ESM Table 10) with a strong focus on hormone secretion (ESM Table 11). Analysis of downstream functions using a literature-based prediction method [31] revealed a decrease in processes controlling cAMP concentrations, neurotransmitter release and synaptic transmission in OD islets, while pathways related to numbers of beta cells, islet cells and neuroendocrine cells were mostly affected in PPP islets (ESM Table 12).

The same method was used to identify putatively activated or inhibited upstream regulators of the regulated genes. For OD islets, the highest inhibition scores were found for *ADCYAP1*, *NEUROD1*, *BDNF* and *PAX6* (ESM Table 13), supporting altered differentiation of beta cells during development and/or function and viability. Significant activation scores were found for *FOXO1* and, in particular, *REST*, two transcriptional regulators, which preclude the differentiation of beta cells and/or the retention of their identity [32, 33]. *HNF1A* was the only transcription factor predicted to be significantly inhibited in PPP islets.

### Analysis of potential upstream regulators revealed key transcription factors involved in beta cell dysfunction

**Fig. 5:**
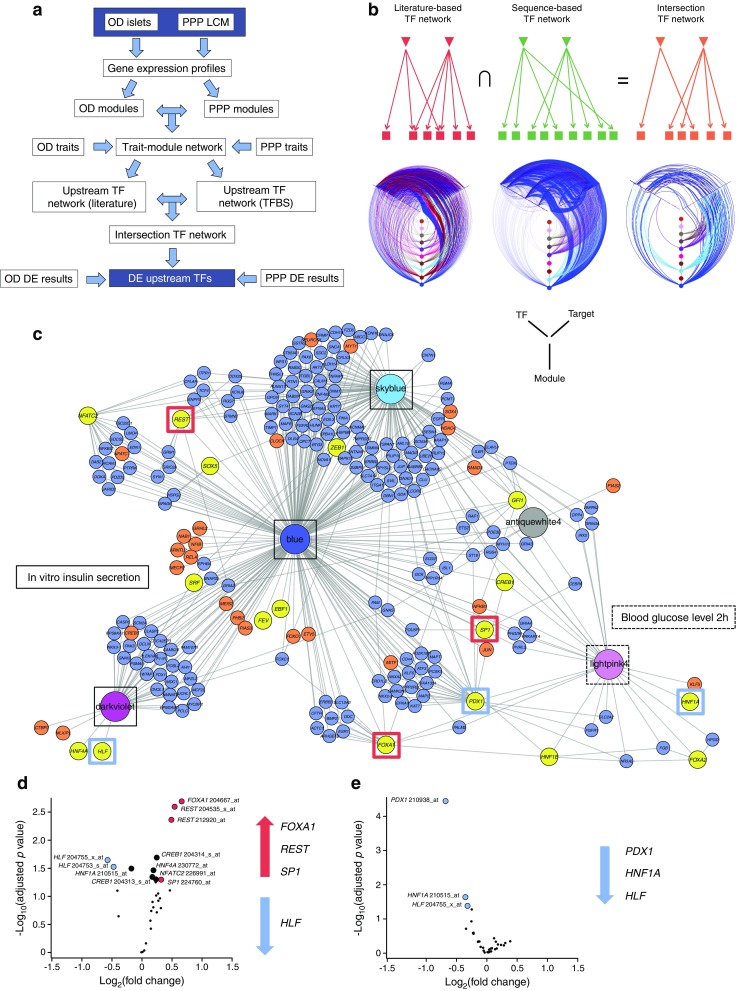
Systems biology analysis predicted the key transcription factors (TFs) regulated in type 2 diabetes. (**a**) Workflow to identify upstream TFs. (**b**) Schematic showing how literature and sequence-based networks were combined to generate an intersection network. TFs are represented by inverted triangles and genes are represented by squares. Evidence for TF gene targets is shown by arrows (literature red, sequence-based green). Intersection Network (orange) shows TF–target gene interactions present in both literature and sequence-based networks. Underneath each schematic is a hive plot showing edges between upstream TFs, target genes and gene co-expression modules. Modules are shown as coloured nodes on the vertical axes. TFs are represented on the left axes and their predicted target genes on the right axes. Edges are coloured according to the gene co-expression module of the source node. Nodes are ordered along the axes by increasing degree from the centre outwards. (**c**) Network representation of the Intersection Network shown in (**b**). Gene co-expression modules are represented as large coloured nodes in the corresponding module colour. skyblue, blue and darkviolet modules are correlated with the insulin stimulation index (ISI) of OD islets (solid black outlines), while lightpink4 module correlates with glucose concentrations at 2 h after an OGTT in PPP islets (dashed box). Yellow nodes indicate potential upstream TFs for each module. Blue or orange nodes indicate putative target genes (TFs in orange). Network edges were predicted by both literature and sequence motif-based approaches. For visualisation, the network was filtered to remove all gene nodes except for TFs with only one edge. For a more detailed view, a PDF version of Fig. 5 is provided as ESM Fig. 11. (**d**, **e**) Volcano plots of differentially expressed TFs in type 2 diabetic vs non-diabetic OD (**d**) and PPP (**e**) islets. Significantly differentially regulated TFs (Limma empirical Bayes adjusted (Benjamini Hochberg method) *p* ≤ 0.05, fold change ≥1.2) are shown as red (upregulated) or blue (downregulated) circles, while potentially differentially regulated TFs with lower fold changes (FDR ≤0.05; no fold change cut-off) are shown as black circles. Upregulated and downregulated TFs are also indicated on the right of each plot. See ESM Table 13 for more details

Gene co-expression modules were identified for non-diabetic OD and PPP islets. Modules that significantly overlapped between OD and PPP were then correlated with clinical or functional traits (see ESM Methods). This identified a set of ten modules in which 14 out of 19 differentially expressed signature genes were enriched (hypergeometric *p* = 3.34 × 10^−5^); only *CAPN13*, *FFAR4*, *NSG1*, *FAM102B* and *KCNH8* were absent from the selected modules. To investigate whether genes within modules share the same transcriptional control, we identified putative upstream transcription factors using two complementary bioinformatics approaches (Fig. 5a). The first used a literature-based prediction method [31], while the second used enrichment of predicted transcription factor binding sites in the promoter sequences of the module genes [34]. The results were then used to generate two networks, one for each analysis, describing predicted upstream transcription factor interactions with their predicted target genes. The networks were merged into a single network containing 17 upstream transcription factors and 29 transcription factor–target gene interactions predicted by both approaches (Fig. 5b, c). Several modules were correlated with insulin and blood glucose levels (Fig. 5c and ESM Figs 6–8).

Of note, three of the 19 differentially expressed signature genes (*PPP1R1A*, *SLC2A2* and *CD44*) were present in this network and were potential targets of PDX1. We therefore hypothesised that PDX1 and other transcription factors in the network regulate the differentially expressed genes in type 2 diabetes. The volcano plots in Figs 5d and e show the 17 transcription factors in OD and PPP islets, respectively. *REST*, *FOXA1* and *SP1* were upregulated and *HLF* was downregulated in OD islets. Meanwhile, *PDX1*, *HNF1A* and *HLF* were downregulated in PPP islets. *HNF1A* tended to be downregulated in OD islets. Other than *HLF*, these transcription factors, are known regulators of beta cell differentiation and function.

### Validation of upstream regulators

**Fig. 6:**
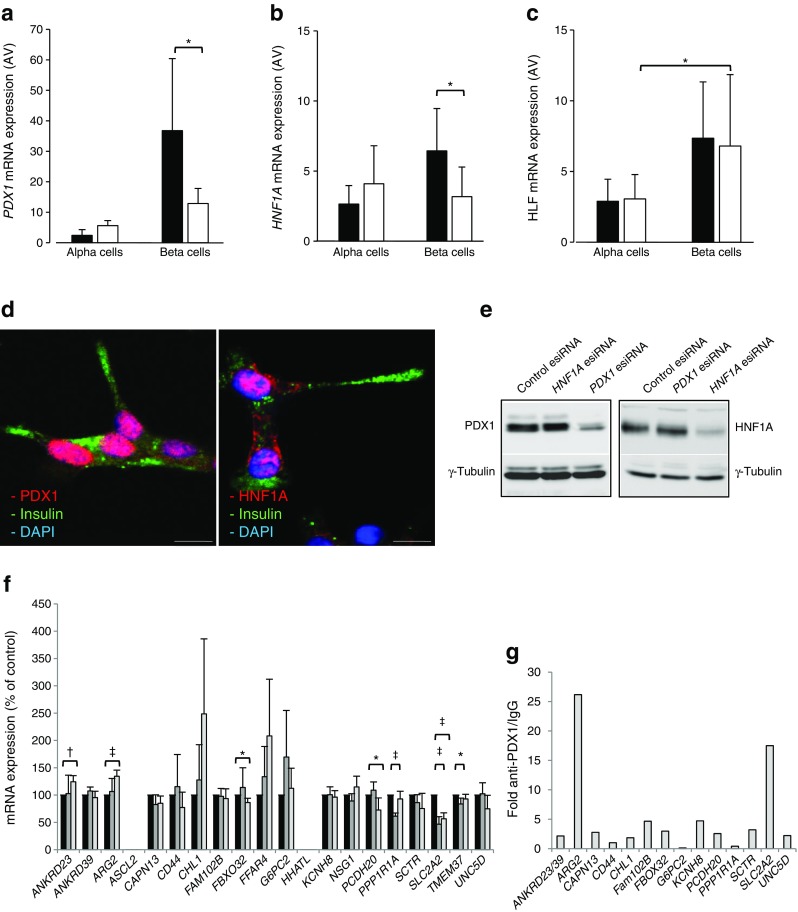
Validation of PDX1, HNF1A and HLF as transcription factors (TFs) located upstream of the T2D islet signature genes. (**a**–**c**) RT-qPCR of *PDX1* (**a**), *HNF1A* (**b**), and *HLF* (**c**) expression levels in alpha and beta cell-enriched fractions from human ND (*n* = 5, black bars) and T2D (*n* = 4, white bars) OD islets. *p < 0.05, Student’s *t* test. (**d**) Co-immunostaining for insulin (green) and *PDX1* or *HNF1A* (red) in human EndoC-βH1 cells. Nuclei are counterstained with DAPI (blue). (**e**) Immunoblots for *PDX1*, *HNF1A* or γ-tubulin in EndoC-βH1 cells treated with esiRNA for *PDX1*, *HNF1A* or with a control esiRNA. Bar, 10 μm. (**f**) RT-qPCR of the 19 T2D islet signature genes in EndoC-βH1 cells treated with esiRNA for *PDX1* or *HNF1A* or with a control esiRNA (*p < 0.05, ^†^ *p* < 0.01 and ^‡^ *p* < 0.001; ANOVA) (*n* = 4, except for *ANKRD39*, which was measured three times). (**g**) Fold enrichment (*y*-axis) of T2D islet signature genes with predicted binding sites for *PDX1* as measured upon chromatin immunoprecipitation with anti-PDX1 antibody vs control IgG followed by RT-qPCR with primers flanking the predicted binding site. The values in (**g**) are from three independent chromatin immunoprecipitations. esiRNA, endoribonuclease-prepared small interfering RNA; ND, non-diabetic; T2D, type 2 diabetic

Among the transcription factors identified above, PDX1, HNF1A and HLF were chosen for further analyses. RT-qPCR assays of human islet alpha and beta cell-enriched fractions confirmed that expression of *PDX1* and *HNF1A* was reduced in type 2 diabetic beta cells (Fig. 6a, b). Although *HLF* was enriched in non-diabetic beta cells, its expression was not altered in type 2 diabetes (Fig. 6c), and its role was not further investigated. Both PDX1 and HNF1A were detected in the nucleus of EndoC-βH1 cells [35] (Fig. 6d). Silencing of *HNF1A* (Fig. 6e, ESM Fig. 9a, b) reduced mRNA levels of *SLC2A2*, *PPP1R1A* and *TMEM37* (Fig. 6f). While *SLC2A2* is a known target of HNF1A [36, 37], *PPP1R1A* and *TMEM37* are not predicted to include a binding site for HNF1A within 5 kb upstream or downstream of their transcription start site (ESM Methods and ESM Table 14). Silencing of *PDX1* reduced *SLC2A2* mRNA levels but increased those of *ANKRD23* and *ARG2* (Fig. 6f). All three genes include one or more putative binding sites for PDX1. Chromatin immunoprecipitation assays did not provide evidence for binding of HNF1A to the promoters of *UNC5D*, *FAM102B* and *CD44*, while the promoter regions of *ARG2* and *SLC2A2*, the latter being a well-established PDX1 target, were recovered with PDX1 (Fig. 6g).

## Discussion

### Rationale and novelty of the methodological approach

We present a novel transcriptomic signature of human type 2 diabetic islets. Our data were obtained from the rigorous analysis of, to date, the largest collection of human islets from non-diabetic (*n* = 116) and type 2 diabetic (*n* = 55) individuals. We exploited two different islet sources (OD and PPP) and islet isolation methods (enzymatic digestion and LCM) to maximise the advantages and circumvent the limitations of each method [38–40]. One concern is that in PPP the underlying pancreatic disease may influence islet cell gene expression. In our PPP cohort, the prevalence of pancreatitis and benign or malignant pancreatic tumours was comparable between the non-diabetic and type 2 diabetic PPP. None of these disorders was associated with islet transcriptome changes (ESM Fig. 10), while PPP and OD islets were equivalent in terms of the presence of ‘contaminating’ acinar and ductal cell markers. The duration of type 2 diabetes in PPP (10.6 ± 8.6 years) and OD (9.9 ± 7.4 years) was comparable and previous studies found no association between diabetes and pancreatic cancer among individuals with long-standing type 2 diabetes [41]. PCA revealed that the transcriptomes of islets isolated from the same organ donor either by enzymatic digestion or by LCM did not cluster, whereas the latter clustered with the transcriptomes of LCM PPP islets. We further compared our results with recently published signatures for four pancreatic cancer subtypes [29] and found that genes differentially regulated in type 2 diabetic islets of OD and PPP showed very similar patterns of enrichment against these signatures (ESM Table 15). These results demonstrate that the transcriptomic differences between OD and PPP samples are not driven by patient differences, but are mainly due to the distinct islet isolation procedures used in each cohort.

For transcriptomic analysis we used microarrays rather than RNA sequencing because when we initiated the sample processing the former offered the most robust and cost-effective solution. In future, it will be valuable to investigate this large set of human islets by RNA sequencing [42], for example, to assess alternative splice variants and the status of non-coding RNA.

In our IGT and type 3c diabetic PPP, as in a previous cohort of type 2 diabetic individuals undergoing surgery for pancreatic cancer [43], glucose intolerance correlated with altered hepatic function and insulin resistance secondary to compression of the biliary duct by the pancreas head [7]. Indeed, equivalent types of tumours in the pancreas body and tail were not associated with glucose intolerance, which ameliorated quickly after removing tumours in the pancreas head [7, 44]. Hence, beta cell dysfunction and hyperglycaemia in IGT and type 3c diabetic PPP are likely to result from the burden that insulin resistance poses on beta cells, rather than from a direct effect of the tumour on such cells.

### Novelty and relevance of the biological findings

None of the 19 signature genes are in any of the established type 2 diabetes-associated genetic loci [45], although *G6PC2* variants affect fasting glucose [46, 47]. The activities of most of them in beta cells are suggested by their associations with islet functional traits and clinical variables. Some of our results confirm previous work showing downregulation of *ARG2*, *CAPN13*, *CHL1*, *FFAR4*, *G6PC2*, *PPP1R1A*, *SLC2A2*, *TMEM37* and *UNC5D* and upregulation of *KCNH8* in type 2 diabetic islets [15, 17, 20]. The involvement of *SLC2A2*, *FFAR4* and *G6PC2* in beta cell function is well established [46, 48, 49], while that of *CHL1* and *PPP1R1A* was reported more recently [15, 17, 50]. Using RT-qPCR and/or immunostaining, we showed that *PPP1R1A*, *ARG2* and *TMEM37* are enriched in human beta cells and their modulation affects insulin release. Sequence similarity suggests that *TMEM37* encodes an evolutionarily conserved inhibitory subunit of voltage-gated calcium channels, but its function has never been validated. We show that its acute depletion increased [Ca^2+^]_i_ levels and insulin secretion upon glucose stimulation, while its overexpression exerted opposite effects. Hence, *TMEM37* reduced expression in type 2 diabetic islets may represent a compensatory effort of beta cells in response to prolonged hyperglycaemia.

Nine genes not previously reported to be associated with type 2 diabetes were significantly dysregulated in our organ donor and PPP cohorts (*ANKRD23/39*, *ASCL2*, *HHATL*, *NSG1*, *PCDH20*, *SCTR*, *CD44*, *FAM102B* and *FBXO32*). Ankyrin repeat domain 23/39 (ANKRD23) is a transcriptional regulator enriched in metabolically active tissues, such as muscle and brown fat [51]. In muscles, it reduces serine/threonine kinase 11 (STK11, also known as LKB1) expression, AMPK phosphorylation [52] and palmitate uptake. Interestingly, its expression is upregulated in insulin target tissues in rat models of type 2 diabetes [51]. Achaete-scute complex homologue 2 (ASCL2) is a transcription factor implicated in fate determination of neuronal precursor cells, while hedgehog acyltransferase-like protein (HHATL) may inhibit N-terminal protein palmitoylation. Their downregulation correlated with de-differentiation of human islets in culture [53]. *ASCL2*, *NSG1*, *PCDH20* and *UNC5D* transcripts are enriched in neurons, which are functionally related to islet endocrine cells. Neuron specific gene family member 1 (NSG1, also known as NEPP21) is implicated in endosomal trafficking of neuronal cell adhesion molecule NRCAM (also termed L1/NgCAM), of which CHL1 is a paralogue, and of glutamate α-amino-3-hydroxy-5-methyl-4-isoxazolepropionic acid (AMPA) and *N*-methyl-d-aspartate (NMDA) receptors. A recent study found that NMDA receptors inhibit insulin secretion [54]. Unc-5 netrin receptor D (UNC5D), like neural cell adhesion molecule L1-like protein (CHL1), may regulate neuronal migration and survival [55] while protocadherin 20 (PCDH20) may inhibit the canonical WNT signalling pathway. Finally, secretin receptor (SCTR) is the most potent regulator of pancreatic bicarbonate, electrolyte and volume secretion. Although early research indicated that secretin stimulates insulin release in humans [56], the role of this hormone and its receptor in beta cell function remains unclear.

Other novel genes upregulated in type 2 diabetic islets were *CD44*, *FAM102B* and *FBXO32*. CD44 is involved in cell adhesion and migration, cell–cell interactions, islet inflammation in type 1 diabetes [57], adipose tissue inflammation, insulin resistance and hyperglycaemia [58]. F-box protein 32 (FBXO32) belongs to the E3-ubiquitin ligase Skp1–Cullin–F-box complex for phosphorylation-dependent ubiquitination. Alterations of the ubiquitin–proteasome pathway are associated with beta cell dysfunction in type 2 diabetes [14].

Our results provide new insights on islets in type 2 diabetes. Upregulation of genes encoding proinflammatory molecules such as *IL1B*, *CCL26*, *CCL3*, *CCL8*, *CXCL1*, *CXCL11*, *CXCL12*, *CXL2* and *CXCR7* in type 2 diabetic OD islets, but not in type 2 diabetic PPP islets, likely reflects a ‘wound’ response secondary to enzymatic isolation [53]. The top genes relevant to beta cell function and downregulated in type 2 diabetic OD islets were *GLP1R*, *IAPP*, *PTPRN*, *ERO1LB*, *ALDOC* and the genes encoding several glutamate AMPA and NMDA receptor subunits, namely *GRIA2*, *GRIA4* and *GRIN2A*. The top upregulated genes in type 2 diabetic PPP islets were the aldolase isoform *ALDOB* and *FAIM2*, while *TMEM27* and *GRIN2D* were downregulated.

Using a novel network-based strategy, we inferred altered activities of *PDX1*, *HNF1A* and *HLF* in type 2 diabetic islets. Both *PDX1* and *HNF1A* were downregulated in enriched beta cell fractions from type 2 diabetic OD, with PDX1 targeting *ARG2*. Downregulation of *HNF1A*, which lies upstream of *PDX1*, and upregulation of *REST*, point to de-differentiation of beta cells in type 2 diabetes. However, this suggestion is tempered by the lack of evidence for upregulation of the so-called ‘disallowed’ beta cell genes [59], as expected in the context of clear de-differentiation. More sensitive approaches, such as RNA sequencing, may be required to detect changes in these weakly expressed genes.

The present results likely represent only the tip of the iceberg in terms of potential targets and biological hypotheses for beta cell alterations in type 2 diabetes. Several other groups have published findings derived from OD islets [11–20]. Since all these studies, including our own, are each likely to capture only part of the ‘truth’, a more complete picture will conceivably emerge from the integration of all available human islet transcriptomic datasets.

An intriguing finding is that none of the 19 signature genes showed significant expression changes in type 3c diabetes or IGT PPP islets. While these findings must be validated in larger cohorts, one implication is that the diverse transcriptomic changes in type 2 diabetic, IGT and type 3c diabetic islets may reflect pathophysiological idiosyncrasies of these conditions and/or the duration and severity of hyperglycaemia. Another possibility is that these transcriptomic changes correlate with beta cell failure but may not precede it. Therefore, islet transcriptomic changes preceding beta cell failure remain elusive.

In conclusion, the present study provides a stringent definition of the transcriptomic signature of a large series of human type 2 diabetic islets, regardless of islet source and isolation procedure. The identification of dysregulated genes, some of them not reported previously, the description of downstream and upstream regulators and the identification of key transcription factors involved in beta cell dysfunction, as reported in this study, contribute to the understanding of the complex molecular scenario of type 2 diabetic islet cells.

## Electronic supplementary material

ESM (PDF 4.36 mb)

ESM Table 7 (XLSX 88 kb)

ESM Table 15 (XLSX 14 kb)

## Abbreviations

AMPA: α-Amino-3-hydroxy-5-methyl-4-isoxazolepropionic acid
ARG2: Arginase 2
[Ca^2+^]_i_: Intracellular Ca^2+^
FDR: False discovery rate
IGT: Impaired glucose tolerance
IMIDIA: Innovative Medicines Initiative for Diabetes: Improving beta-cell function and identification of diagnostic biomarkers for treatment monitoring in Diabetes
HNF1A: HNF1 homeobox A
LCM: Laser capture microdissection
MODY: Maturity onset diabetes of young
NMDA: *N*-methyl-d-aspartate
OD: Organ donors (cohort)
PCA: Principal component analysis
PDX1: Pancreatic and duodenal homeobox 1
PPP: Phenotyped pancreatectomised patients
PPP1R1A: Protein phosphatase 1 regulatory inhibitor subunit 1A
RMA: Robust multi-array average
RT-qPCR: Reverse transcription quantitative PCR
TMEM37: Transmembrane protein 37
