# Supplementary material for: Systems biology of the IMIDIA biobank from organ donors and pancreatectomised patients defines a novel transcriptomic signature of islets from individuals with type 2 diabetes
Source: Diabetologia. 2017 Nov 28;61(3):641–57. doi: 10.1007/s00125-017-4500-3 (PMC5803296; doi:10.1007/s00125-017-4500-3)
Supplement: Supplementary file 1 — (PDF 4.36 mb) [file 125_2017_4500_MOESM1_ESM.pdf]

ESM Methods

Islet procurement, insulin secretion and RNA extraction

Islet procurement from OD. Pancreases not suitable for transplantation were obtained in Pisa from 161 non-diabetic and 39 type 2 diabetic heart-beating OD under approval from the local ethics committees (ESM Table 6). The donors' full clinical history and major laboratory parameters, such as blood glucose during the intensive care unit stay and fructosamine, were collected to assess the presence of diabetes. Well-preserved islets were isolated from the pancreases of 153 non-diabetic and 34 type 2 diabetic OD. After 2±1 days of culture, islets were successfully hand-picked from 141 preparations (115 ND and 26 type 2 diabetic) and processed for further analyses. Forty-three human islet preparations, all from non-diabetic OD, were acquired by Eli Lilly from Prodo Laboratories Inc. (Irvine, CA, USA). Isolated islets were prepared by enzymatic digestion and were shipped to Lilly after recovery for 1–2 days in culture.

Islet procurement from PPP. Pancreatic tissues and blood samples were collected from patients undergoing partial pancreatectomy in the Department of Surgery, University Hospital of TU Dresden. Patients age <18 years were excluded. The local ethics committee approved the study and all of the patients provided written informal consent. Information collected immediately before surgery included: individual and family medical history, BMI, standard clinical parameters, HbA_{1c} concentrations, and fasting glucose and insulin concentrations. Non-diabetic subjects with fasting glycaemia <7.0 mmol/l underwent an OGTT to measure glucose, insulin, proinsulin and C-peptide at 0, 60 at 120 min after a glucose load within a few days before surgery. Among PPP 70 were ND (fasting glycaemia <7.0 mmol/l; HbA1c <6.5%, glycaemia at 2 h after presurgical oral glucose tolerance test [OGTT] <7.8 mmol/l), 54 had T2D (fasting glycaemia ≥7.0 mmol/l; HbA1c ≥6.5%, history of diabetes for >1 year), 30 had impaired glucose tolerance (IGT) (fasting glycaemia <7.0 mmol/l; HbA1c <6.5%, OGTT at 2 h of ≥7.8 to <11.1 mmol/l), and 46 had diabetes, likely due to the associated pancreatic disorder leading to surgery, also termed type 3c diabetes (T3cD) (ESM Table 6). A diagnosis of T3cD was made if pathological glucose tolerance (fasting glycaemia ≥7.0 mmol/l, HbA1c ≥6.5% and/or pre-surgical OGTT at 2 h ≥11.1 mmol/l) was first detected <1 year before the symptoms, which led to surgery. Fasting potassium and magnesium concentrations were measured to exclude false-positive results. All patients were screened for autoantibodies against insulin, GAD65, ZnT8, and IA-2/ICA512, as previously described [1].

Insulin secretion from OD islets. Islet insulin release was assessed by immunoradiometric assay, as previously described [2, 3]. After isolation, the islets were cultured for 2–3 days before groups of 15 islets of comparable size were hand-picked and incubated at 37°C for 45 min in Krebs–Ringer bicarbonate solution (KRB), 0.5% albumin, pH 7.4, and 3.3 mmol/l glucose. Then, the medium was completely removed, assayed for basal insulin secretion, and replaced with KRB containing either 16.7 mmol/l glucose, 3.3 mmol/l glucose plus 100 μmol/l glyburide, or 3.3 mmol/l glucose plus 20 mmol/l arginine. After incubation for 45 min, the medium was removed and insulin levels were measured to assess stimulated insulin release. Insulin secretion was also expressed as insulin stimulation index (ISI) – the ratio of stimulated to basal insulin secretion.

Extraction of RNA from islets isolated enzymatically or by LCM. Two–three days after enzymatic isolation, groups of 100–120 islets from 125 OD at Univ. Pisa were hand-picked, rinsed in sterile PBS (Sigma-Aldrich) and centrifuged at 3,000 × *g* for 5 min. The supernatant was disposed and 100 µl of extraction buffer (PicoPure RNA Isolation Kit; Life Technologies, Foster City, CA) was added. Samples were incubated at 42°C for 30 min, centrifuged at 3,000 × *g* for 2 min, and the supernatant was collected and stored at −80°C until RNA isolation. RNA isolation was performed according to the manufacturer's protocol. Briefly, 100 µl of 70% ethanol were added to the cell extract, the mixture was added to the purification columns, washed, and subjected to DNase treatment by incubation with 40 µl of DNase I solution (RNase-Free DNase Set; Qiagen GmbH, Hilden, Germany) for 15 min. Two additional washes were performed and RNA was eluted in 30 µl of elution buffer. The RNA concentration and purity were evaluated using a NanoDrop 2000c spectrophotometer (Thermo Scientific, Wilmington, DE) and RNA quality was evaluated using the Agilent 2100 Bioanalyzer system and the Agilent RNA 6000 Pico Kit (Agilent Technologies, Santa Clara, CA). The same procedure was applied at Lilly, Indianapolis for RNA extraction from islets of 39 OD. The mean yield of total RNA from 100–200 islets from non-diabetic and type 2 diabetic subjects at University of Pisa was 913±340 ng (RNA Integrity Number [RIN]: 8.1±0.5) and 1016±329 ng (RIN: 8.3±0.4), respectively. In the case of islets processed at Eli Lilly in Indianapolis, the mean yield of total RNA from 5,000 islet equivalents was 7.13±6.61 µg with a RIN of 8.0±0.5.

Pancreatic islets within resected pancreas specimens were isolated in Dresden by LCM with a Zeiss Palm MicroBeam system in the light microscopy facilities of the BIOTEC/CRTD at TU Dresden and MPI-CBG. In the case of three PPP islets were also isolated in parallel from small surgical specimens as previously described [4], except for the use of MTF as protease blend (Roche, Penzberg, Germany). Conversely pancreatic tissue sections from three OD whose islets were isolated eyzmatically in Pisa were used as a source for islet retrieval by LCM in Dresden, following the same protocol applied for LCM of islets for surgical specimen of PPP. RNA extraction from LCM islets was also performed using the PicoPure RNA Isolation Kit as previously described [5]. Information about average islet yield, RNA quantity and RIN is provided in ESM Table 16.

Preparation of human islet beta and alpha cell-enriched fractions. The methods used to prepare the human islet beta and alpha cell-enriched fractions were previously described [6] and validated [7,8]. Briefly, human islets were dissociated into single-cell suspensions by incubation with constant agitation for 3 min at 37°C in 0.05% trypsin-EDTA (Thermo Fisher Scientific Inc.) supplemented with 3 mg/ml DNAse I (Roche, Basel, Switzerland), followed by pipetting vigorously to complete the dissociation. Labelling and sorting of the alpha and beta cell fractions was performed by Newport Green labelling [9] followed by FACS, as previously described [8].

Microarrays

RNAseq of OD islets exposed ex vivo to hyperglycaemia.

Three independent islet preparations from ND OD (age: 80±4 years, sex: 1F/2M, BMI: 22.7±0.6 kg/m^2) were used to assess islet gene expression after exposure to 22.2 mmol/l glucose. Briefly, after isolation the islets were cultured for 2 days in M199 culture medium with 5.5 mmol/l glucose; then batches of islets were cultured for additional 2 days in medium with either 5.5 mmol/l glucose (control islets) or 22.2 mmol/l glucose. At the end of the culture period,

approximately 120 islets hand-picked and their RNA extracted using the RNeasy Mini Kit plus QIAshredder (Qiagen, Hilden, Germany) through lysing and homogenizing steps followed by DNA digestion using RNase-Free DNase Set (Qiagen) and washing steps. Total RNA concentration was measured using the NanoDrop™ 2000c Spectrophotometer (Thermo Fisher Scientific, Waltham, MA USA) and RNA quality was assessed by Agilent Bioanalyzer 2100 Instrument (Agilent Technologies, Wokingham, UK) and Agilent RNA Nano Chips (Agilent Technologies). The mean yield of total RNA was 1,298±374 ng and the mean RIN was 8.4±0.7.

RNA quality assessment, processing, and transcriptomic profiling. Total RNA of islets from OD and PPP was quantified with the NanoDrop 2000 Spectrophotometer (Thermo Scientific). RNA quality was assessed using the Agilent 2100 Bioanalyzer and the Agilent RNA 6000 Pico kit (Agilent Technologies) and further processed for Affymetrix if the RIN was >4.1. For cDNA preparation, 10–25 ng of total RNA was amplified using the Ovation RNA Amplification System V2 and was subsequently labelled with Biotin using the Encore™ Biotin Module according to the manufacturer's instructions (NuGEN Technologies, San Carlos, CA). The length distribution of the amplified cDNA products was determined using an Agilent 2100 Bioanalyzer (Agilent Technologies). Hybridisation of biotin-labelled cDNA to Affymetrix HG_U133Plus2.0 GeneChip microarrays was performed at ATLAS Biolabs GmbH (Berlin, Germany). Briefly, 3.75 µg of cDNA per sample were hybridised to the genechips for 16–18 h at 45°C in a rotating hybridisation oven at 60 rpm (Hybridization Oven 640; Affymetrix). The array was subsequently washed and stained with a fluidics station (GeneChip® Fluidics Station 450; Affymetrix) according to the EukGe_WSv4_450 fluidics protocol for eukaryotic 3'-expression arrays. Arrays were scanned using GeneChip Scanner 3000 7G (Affymetrix) and primary data analysis was performed with Affymetrix software GeneChip Operating System (GCOS) v1.4.

Cell culture and transfection. CHO cells grown on SuperFrost Plus slides were transiently transfected with cDNA vectors using Lipofectamine according to the manufacturer's instructions (Thermo Fisher Scientific Inc.). Cells were fixed for 24 h after transfection with formalin for 30 min and then used for validation of anti-ARG2 (sc-20151, Santa Cruz Biotechnology, Dallas, TX), anti-PPP1R1A (ab40877, Abcam, Cambridge, UK) or anti-TMEM37 antibodies (ab111287, Abcam) for immunocytochemistry using the Ventana XT staining system (Roche). Cell transfection was verified in parallel by immunocytochemical detection of overexpressed GFP. Silencing of *PDX1* and *HNF-1A* in EndoC-βH1 cells [10] was achieved using 50 pmol of esiRNA (Eupheria, Dresden, Germany) with Dharmafect4 as the transfection reagent (GE Dharmacon, Lafayette, CO).

INS-1 832/13 cells were cultured in a humidified atmosphere containing 5% CO_2 in complete RPMI 1640 medium (Thermo Fisher Scientific Inc.) supplemented with 10% heat-inactivated fetal bovine serum, 1 mmol/l sodium pyruvate, 50 µmol/l 2-mercaptoethanol, 2 mmol/l glutamine, 10 mmol/l HEPES, 100 U/ml penicillin, and 100 µg/ml streptomycin. Silencing of *PPP1R1A*, *ARG2* and *TMEM37* in INS-1 832/13 cells was achieved by transfection with the respective siRNAs and Lipofectamine 3000 diluted in Opti-MEM. After incubation overnight, the cells were cultured in RPMI medium for 24 h. For cytoplasmic Ca^{2+} imaging experiments, INS-1 832/13 cells were co-transfected with the fluorescent transfection marker siGLO RISC-Free siRNA to identify siRNA-transfected cells. For overexpression of *TMEM37-V5*, INS-1 832/13 cells were transfected with the corresponding pcDNA3.1 vector using Lipofectamine 2000. Four days after transfection, cells were harvested for immunoblotting and

immunocytochemistry. For Ca^{2+} imaging analysis, *TMEM37-V5* INS-1 832/13 cells were identified by co-transfection with pEGFP-C1 (Takara Bio Inc., Kusatsu, Japan).

cDNA vectors. The open reading frames for human *ARG2* (BCA001350), *PPP1R1A* (NM_006741), and *TMEM37* (XM_005263597, NM_019432) were cloned into pcDNA3.1neo_DEST. Human *TMEM37* was also cloned into pcDNA3.1neo_3cMYC_DEST, while mouse *Tmem37* (NM_019432) was inserted into pcDNA3.1D/V5-His-Topo according to the manufacturer's instructions (Thermo Fisher Scientific Inc.). The forward and reverse primers are shown in ESM Table 1.

Ca^{2+} imaging. INS-1 832/13 cells were grown on glass coverslips. The wide-field imaging system consisted of an Olympus IX70 multi-parameter fluorescence microscope, monochromator, and objective lens (×40/1.35NA oil UApo/340; Olympus). All imaging experiments were performed at 37°C in a heated perfusion chamber using pre-bubbled imaging solution (in mmol/l: 140 NaCl, 3.6 KCl, 0.5 NaH_2PO_4, 0.5 $MgSO_4$, 1.5 $CaCl_2$, 10 HEPES and 2 $NaHCO_3$, pH 7.4). The basal and loading solution contained 3 mmol/l glucose, essentially as previously described [11]. In imaging solution containing 20 mmol/l KCl, NaCl was reduced to 123.6 mmol/l (3 mmol/l glucose). Cytoplasmic (intracellular) free Ca^{2+} levels ($[Ca^{2+}]_i$) were recorded using ratiometric Ca^{2+}-sensitive dyes. Cells were loaded for 40 min at 37°C with either 4 µmol/l Fura Red or 4 µmol/l Fura-2 AM in imaging solution containing 0.004% Pluronic (Thermo Fisher Scientific Inc.). After loading, the cells were washed in basal solution for ≥20 min to allow de-esterification of the dyes. Images were acquired using Micromanager software at a rate of 1 frame/3 sec. Cells were excited at 420 and 480 nm for Fura Red loaded cells and at 340 and 380 nm for Fura-2 AM loaded cells. The emitted light was passed through an ET525/50 filter equipped with a T495lpxr dichroic mirror for Fura-2 AM or an ET630/75 filter equipped with a T585lpxr dichroic mirror for Fura Red (Chroma, Bellows Falls, VT). Cells with responses of ≥3 standard deviations higher than the averaged baseline to both KCl and glucose stimuli and with an increase of ≥10% after an increase to 15 mmol/l glucose were considered responsive and were included in the analysis. The basal cytosolic Ca^{2+} concentration was calculated as the mean of the ratios measured over the first 150 s. The maximal fluorescent intensity value was taken as the peak amplitude. To calculate the cumulative fluorescence, curves were first smoothed using a fast Fourier transform filter and the baseline was subtracted. Then, the area under the curve was derived via integration.

RT-qPCR. Total RNA was extracted from human dispersed, FACS-sorted islet cells, rat INS-1 832/13 cells, and human EndoC-betaH1 cells using RNAeasy kits according to the manufacturer's instructions (Qiagen) and then reverse-transcribed using Superscript Reverse Transcriptase II (Thermo Fisher Scientific Inc.). RT-qPCR was performed using GoTaq qPCR Master Mix (Promega, Madison, WI). The mRNA levels of the genes of interest in human islet dispersed cells were quantified and normalised to β-actin in a VIIA7 system (Applied Biosystems). The gene expression levels in INS-1 832/13 cells, EndoC-betaH1 cells and human islets were normalised to β-actin expression. The forward and reverse primers purchased from commercial suppliers are indicated in the table below.

In situ RT-PCR. Pancreatic sections from 10 non-diabetic and 10 age-matched type 2 diabetic OD and from 10 non-diabetic and 10 age-matched type 2 diabetic PPP were placed on three-

chamber slides. Briefly, after dewaxing and different pre-incubation steps, reverse transcription was performed with oligo-dT primers (Invitrogen), M-MLV reverse transcriptase (0.5 U/µl) (Invitrogen), and RNasin (Promega) for 1 h at 37°C in a humidified chamber. After inactivation of the reverse transcriptase, 15 µl of PCR reaction mix containing 5% digoxigenin (DIG)-11-dUTP, the mRNA-specific primers and selfseal-reagent (BioRad) was applied to each chamber of the slide. The slides were sealed with coverslips and were placed into a PTC-200 DNA Engine thermal cycler with a slide block (BioRad, Munich, Germany) for *in situ* RT-PCR, as previously described [12, 13]. Amplification for 12 (*ARG2, ASCL2*, *CHL1 [CHL1iso-1*; *CHL1iso-2*, *CHL1-iso3]*, *FAM102B*, *FFAR4A*, *HHATL*, *KCNH8*, *PPP1R1A*, *SCTR*, *SCL2A*, *TMEM37* and *UNC5D)* out of the 19 signature genes of type 2 diabetic islets was performed according to the following protocol: initial denaturation at 95°C for 3 min, followed by 35–40 cycles with a denaturation step at 95°C for 45 sec, annealing at 57°C for 45 s and extension at 72°C for 45 s, followed by a final extension for 10 min at 72°C. The primers are indicated in ESM Table 3. After a blocking step, the incorporated DIG-labelled nucleotides were detected by incubation with ABC-AP goat IgG and a goat anti-digoxigenin antibody (Enzo Life Sciences, Lörrach, Germany) (1:500) at room temperature for 1 h. Slides were developed with nitroblue tetrazolium chloride/5-bromo-4-chloro-3-indolyl phosphate toluidine salt for 1 h at room temperature and counterstained with haematoxylin.

Immunoblotting. The protein concentration of each extract was determined using the BCA assay (Thermo Fisher Scientific Inc.). Extracted proteins (20 µg/lane) were separated on 12% SDS-PAGE and transferred onto polyvinylidene difluoride membranes (GE Healthcare Europe, Little Chalfont, UK). Membranes were probed with mouse monoclonal anti-V5 (R960-25, Thermo Fisher Scientific Inc.), mouse monoclonal anti-gamma-tubulin (T6557, Sigma-Aldrich), rabbit polyclonal anti-PDX1 (07-696, Merck Millipore, MA), and rabbit polyclonal anti-HNF1A (ab174653, Abcam).

Immunomicroscopy. Human pancreatic samples from OD were taken before islet isolation, fixed in 4% paraformaldehyde and embedded in paraffin, essentially as previously described [14]. Surgical pancreatic specimens were immediately fixed after collection with 4% paraformaldehyde in PBS and embedded in paraffin. For cryosections, small tissue fragments were embedded in TissueTek, snap frozen, and stored at −80°C. Formalin-fixed, paraffin-embedded tissue sections (2–3-mm-thick) were stained with the following primary antibodies: guinea pig polyclonal anti-insulin (ab7842, 1:1,000), rabbit monoclonal anti-PPP1R1A (ab40877, 1:200), and rabbit polyclonal anti-TMEM37 (ab111287, 1:30) (all from Abcam); mouse monoclonal anti-glucagon (G2654, 1:3,000), and rabbit polyclonal anti-ARG2 (1:25) (both Sigma-Aldrich). After adding fluorochrome-conjugated secondary antibodies, the sections were imaged using a Leica 1DM5500B microscope (Leica, Wetzlar, Germany) or a Scanscope FL instrument (Roche) to image the TMEM37 staining profile. EndoC-betaH1 cells grown on coverslips were fixed with 4% paraformaldehyde and immunostained with the following primary antibodies: mouse monoclonal anti-insulin (I2018, Sigma-Aldrich), guinea pig polyclonal anti-PDX1 (ab47308, Abcam), and rabbit polyclonal anti-HNF1A (A304-052A, Bethyl Laboratories, Montgomery, TX). Nuclei were counterstained with DAPI (Sigma Aldrich), and coverslips were mounted with Mowiol (Calbiochem/EMD Millipore, Darmstadt, Germany). Images of 0.5-µm-thick optical sections were acquired at room temperature with an inverted confocal microscope (Zeiss Axiovert 200M) equipped with a Plan-Apochromat ×63 oil objective (numerical aperture

1.4), a Zeiss LSM510 scan head with photomultiplier tubes, and analysed using Zeiss LSM 510 AIM software version 4 (Zeiss, Göttingen, Germany).

Chromatin immunoprecipitation. 2×10^7 EndoC-betaH1 cells grown in T75 flasks were cross-linked with 10 ml of 1% formaldehyde for 30 min and then quenched with 1.25 ml 1M glycine for 10 min. Cells were resuspended in nuclear lysis buffer and sonicated with 20 pulses of 20 s each with breaks of 20 s between each sonication cycle to achieve fragments ranging in size between 200 and 2,000 bp. The sonicated cells were diluted 10-fold, centrifuged for 10 min at 13,000 × *g*. Rabbit polyclonal anti-PDX1 (Merck Millipore) and anti-HNF1A (Abcam) antibodies and control rabbit IgG (Dianova, Hamburg, Germany) were used at 10 µg/ml for ChIP analysis. Purified DNA was analysed by RT-PCR (Aria Mx cycler, Agilent Technologies) using 1 µl of DNA as template in 20 µl reaction carried out in triplicate. The primer concentration was 100 nM and their sequences are indicated in ESM Table 4.

Data analysis

Normalisation and statistical analysis of microarray data. The transcriptomics data were summarised and normalised by Robust Multi-array Average (RMA) in Array Studio software (Omicsoft Corp., Cary, NC). Batch correction of microarray data was performed using the R/Bioconductor package ComBat [15, 16]. Elimination of technical outlier samples was performed at two steps of the transcriptomics analysis (Fig. 5a). Briefly, before batch correction and statistical analysis, the array data were filtered to detect genes showing significant expression. The criterion for expression was an intensity value of >75% for ≥25% of the samples in that group. Subsequently, islet samples from OD with no previous history of diabetes but with high blood fructosamine (>285 µmol/l) or glucose (>11.1 mmol/l) were excluded from the analysis to avoid the impact of confounding factors, such as hyperglycaemia caused by the patient's stressful clinical situation. Islet samples from non-diabetic and type 2 diabetic OD with insulin expression levels below one standard deviation from the within-group mean were also eliminated. Covariate correction for age and sex, as well as the statistical analyses, were performed using the linear modelling features of the R/Bioconductor package Limma [17]. In comparisons between type 2 diabetic OD and non-diabetic OD islets, significant differences were defined as a change in expression of ≥1.5 after correction for multiple hypothesis testing using the Benjamini–Hochberg method ($p\leq0.05$). Principal component analysis was performed using the R package prcomp. The analysis of all islet sample types and of a single sample type (islets from OD or PPP) was based on the intensities of the probe sets after batch correction. Contamination of islet samples with exocrine pancreatic tissue was determined using selected markers of exocrine and ductal cells, as indicated in ESM Table 5. A meta-analysis against pancreatic cancer signatures was then performed, as described in ESM results.

Gene ontology overrepresentation analysis of differentially expressed genes. The Bioconductor package GOstats [18] was used to test for the association between the biological process gene ontology terms and the lists of differentially expressed genes in islets of OD and PPP. For differentially expressed genes, a FDR threshold of 0.05 and an absolute fold-change threshold of 1.5 were used. A significance threshold of $p\leq0.05$ and a minimum number of three genes per term were used in the gene ontology-based over-representation analyses.

Enrichment of genes involved in insulin secretion. Enrichment analyses of upregulated and downregulated genes were performed against gene ontology categories for OD islets (208 upregulated and 400 downregulated probe sets) and PPP islets (128 upregulated and 80 downregulated probe sets). Statistical significance was calculated using a one-sided Fisher's exact test.

Pathway analysis of differentially expressed genes. Putatively active pathways, downstream processes, and upstream regulators were determined by IPA (Ingenuity® Systems) (18). A FDR threshold of 0.05 and an absolute fold-change threshold of 1.5 were used to identify differentially expressed genes in OD islets. The absolute fold-change threshold was relaxed to 1.2 to identify differentially expressed genes in PPP islets. The enriched canonical pathway analyses were performed with a threshold of $p<0.05$. Only pathways containing at least three regulated genes were considered. An absolute z-score threshold of 2 and a threshold $p<0.05$ were applied to predict increased/decreased downstream processes and activated/inhibited upstream regulators [19].

Prediction of binding sites for HNF1A and PDX1 in type 2 diabetic islet signature genes. Type 2 diabetic islet signature genes were searched for the inclusion of putative binding sites for HNF1A and PDX1 within 5 kb upstream and downstream of their respective transcription start site using oPOSSUM 3.0 (ESM Table 14).

Identification of gene co-expression modules from OD-islet and PPP-LCM samples. Module generation was performed using Weighted Gene Correlation Network Analysis (WGCNA) [20]. With WGCNA, weighted correlations are calculated between genes based on the gene's expression in the different samples; a weighted correlation is a correlation raised to a certain power (called the soft thresholding power). In WGCNA, the weighted correlations are used to create a specific type of network (a topological overlap network). This network is based on network topology rather than direct correlations between genes: genes are strongly linked together in this network if they share many correlated neighbours with each other. This topological overlap network is then analysed to identify network modules of genes that are strongly linked together. Identifying network modules from the gene expression in this way focuses on groups of genes that are predicted to be functionally related based only on the gene expression data.

WGCNA was performed on genes from 84 OD-Islet and 32 PP-LCM samples from non-diabetic subjects. The non-diabetic samples were chosen so that the gene-gene correlations would not be driven by differences between type 2 diabetic and non-diabetic sample groups and would rather reflect putative functional links between genes. Normalised expression data was first batch corrected using *ComBat* [16] and corrected for age and sex effects by linear regression using *LmFit* from the limma package in R [17]. Both sets of data were then filtered by calculating a co-variance matrix and removing the 25% least co-variant genes. Co-expression networks were constructed by calculating adjacency matrices for each data set using a soft-thresholding power of 7 and Spearman correlation using pairwise complete observations. A topological overlap matrix (TOM) was then calculated from each adjacency matrix, converted to distances, and clustered by hierarchical clustering using average linkage clustering. Modules were identified by dynamic tree cut with a cut height of 0.995 with a minimum module size=20, using the hybrid

method. Module *eigengenes* were calculated and similar modules were merged together using a module *eigengene* distance of 0.15 as the threshold.

Generation of a trait module network linking OD-islet with PPP-LCM modules. Since one of the main goals of our study was to investigate type 2 diabetes-related genes in two different sample collections, we measured similarities at the level of the gene co-expression modules. To do this we compared each OD-islet with each PPP-LCM module pairwise and calculated the significance of enrichment using a one-sided Fisher's exact test. ESM Fig. 12 is a heatmap showing the pairwise module overlaps. Red indicates that a pair of modules has a significant number of overlapping genes. From the figure, it is clear that a number of modules significantly overlap between the two sample types.

We next correlated the *eigengenes* (the first principal components) of the modules to clinical and functional traits specific to each sample type using Spearman correlation (pairwise complete observations) and calculated corresponding p-values for the correlations using the *cor.test* function in R. The traits that were correlated to the module *eigengenes* are shown in ESM Table 17.

We identified the modules that were most correlated with the traits by applying an unadjusted cutoff of $p \leq 0.05$. These module-trait correlations were then plotted as separate heatmaps for OD and PPP modules (ESM Figs. 11, 6-8).

The module-module overlaps and the module-trait correlations were combined together into a module-trait network, where modules and traits were represented as nodes and module-module enrichment scores (−log10 p-value) and module-trait correlations represented the edges (ESM Fig. 8). The network was constructed by creating an edge between two modules if the p-value (adjusted) for enrichment was ≤0.05, or between a module and a trait if the module-trait correlation was considered as significant (unadjusted p-value ≤0.05). The largest connected component in this network comprised a set of 10 modules (4 from OD islets and 6 from PPP islets) that were connected to each other either because the modules were significantly overlapping, or because the modules were correlated to the same traits within the OD and PPP datasets (ESM Fig. 11). These modules were used as a starting point for further analysis described below.

Significance of gene co-expression modules. We tested the significance of the co-expression modules by performing a bootstrap test as follows. For each selected module of size *N*, we randomly sampled *N* probe sets from the expression data 1000 times and calculated the intramodular connectivity [19] (the connectivity of nodes to other nodes within the same module) of the probe sets. To measure the degree of connectivity within the modules compared to the background, we calculated a Z-score as:

$$Z = \frac{k - \mu}{\sigma}$$

where k is the intra-modular connectivity and μ and σ are the mean and standard deviation of intramodular connectivity from 1000 randomly sampled modules of size *N* respectively. Empirical p-values were also calculated as the fraction of background intramodular connectivity

scores greater than or equal to the intramodular connectivity for a particular module. All of the background intramodular connectivity scores were below the module intramodular connectivity for the selected modules.

Significance of selected OD and PPP modules. The Z-score is a measure of the number of standard deviations of the intramodular connectivity of probesets within a module above a background model based on 1000 samples of the same size. The results in the ESM Table 18 show that all of the selected modules have high Z-scores, indicating that it is extremely unlikely to find modules of the same size and connectivity by chance from the expression data

Identification of module hub genes and measuring overlap with signature genes. A hub gene is a highly connected gene within a module that could be influencing a particular trait. Such genes might therefore represent key genes that could have a stronger influence on a trait compared to other genes. Hub genes were selected for each significantly correlated module/trait pair in the module-trait network (ESM Fig. 8) as those that correlated with the modules *eigengene*, the first principal component of the module (Spearman's $r \geq 0.6$), and the trait (Spearman's $r \geq 0.3$). We tested the hub genes for enrichment of the 19 genes differentially regulated in both OD and PPP cohorts using the hypergeometric distribution in R (hyperg function) with the background number of genes equal to the union of the numbers of expressed genes in OD and PPP cohorts (N=15716). The total number of module hub genes was 4285. We found 15 of the 19 signature genes were hub genes in these modules and this is statistically significant (hypergeometric $p=3.34\times10^{-5}$). These signature 'hub' genes are: *ARG2*, *CHL1*, *PPP1R1A*, *CD44*, *HHATL*, *ANKRD23*/*39*, *ASCL2*, *UNC5D*, *PCDH20*, *FBXO32*, *SCTR*, *G6PC2*, *TMEM37* and *SLC2A2*. Four of the signature genes *CAPN13*, *FFAR4*, *NSG1* and *FAM102B* were not module 'hub' genes.

Generation of a sequence-based transcription factor network. We reasoned that the hub genes could also be targets for transcription factors (TFs) affecting a particular trait, since these genes are both highly connected to other module genes and are also correlated to glycaemia-related traits. In order to identify possible TFs upstream of the modules we analysed the promoters of the 4285 module hub genes for the presence of transcription factor binding sites (TFBS) using oPOSSUM 3.0 single site analysis [21]. The promoter regions used in the analysis were from 2000 nucleotides upstream of the TSS to the TSS of each gene. All vertebrate JASPAR profiles with a minimum specificity of 8 bits were used for the analysis. TFs were selected showing significant binding site enrichment measured by both empirical Z-score and Fisher score cutoffs (Z-score ≥10 AND Fisher score ≥7).

The oPOSSUM results for each significantly enriched TF were parsed to create a directed network of TFs and their predicted targets. The TFs were also analysed separately using oPOSSUM to detect potential binding events between TFs (ESM Table 19). Here, no Z-score or Fisher score cutoff was used because the goal was not to test for enrichment, but rather to identify potential TF–TF regulation. A directed network was created for each module-trait combination with genes as nodes and edges between nodes representing evidence of binding-site prediction. Subsequent network analysis was performed in *R* using *iGraph* [22].

Generation of a literature-based transcription factor network. Literature-based networks were created for the same module-trait gene sets using Ingenuity upstream regulator analysis [19]. To do this, significant of overlap between the gene sets and known targets of transcriptional regulators from the literature were calculated using Fisher's exact test. Significant regulators were identified as those with an overlap *p*-value ≤0.01. The predicted upstream regulators were then filtered to select only those that were transcription factors. A directed network was created for each module-trait combination with genes as nodes and edges between nodes representing predicted transcriptional regulation. Subsequent network analysis was performed in *R* using *iGraph* [22].

Merging of sequence-based and literature-based Transcription Factor networks. We reasoned that TF-target gene relationships that were predicted by both a sequence-based and a literature-based approach would constitute robust predictions of upstream regulators of the trait-associated modules. We therefore created a network combining the information from the literature-based and sequence-based networks. To do this, the individual module networks were merged to create a single literature-based, and a single sequence-based network by taking the union of all of the edges in the individual module networks. The literature and sequence-based networks were then combined to create a network containing only the intersecting edges. Hive plots [23] (Fig. 5b) were created for the sequence-based, the literature-based and the intersection network using the *HiveR* package in R (https://github.com/bryanhanson/HiveR). Network visualisation (Fig. 5c) was performed using Gephi (http://gephi.github.io/).

Processing and analysis of RNA-Seq data from islets exposed ex vivo to hyperglycaemia.
Single end reads (75 bp) were aligned on hg19 using tophat and bowtie2 (v 2.0.11 and v. 2.2.1, respectively) and using samtools (v. 0.1.19) for sorting of alignment files. Read counts per gene were then generated using htseq-count (v. 0.5.4p3) and GRCh37.75 Ensembl annotation. Differentially expressed genes were detected using two different methods: DESeq2 and Limma in R. For DESeq2, single end reads (75 bp) were aligned on hg38 using GSNAP (v2017-03-17) and Ensembl annotation 87 was used to detect reads spanning splice sites. The uniquely aligned reads were counted with featureCounts (v1.5.2) and the same Ensembl annotation. The raw counts were normalised based on the library size and testing for differential gene expression between the two conditions, samples treated with glucose versus control, was performed with the DESeq2 R package (v1.15.51). For Limma, the raw count data were first filtered for an average of at least 5 reads in all the samples, normalised to library size using edgeR (v. 3.16.5) TMM method, and then transformed to log2-cpm (counts per million reads) using the *voom* function in R. Empirical Bayes moderated t statistics and corresponding *p*-values were then computed comparing the samples treated with glucose to controls using the Limma package. The *p*-values were adjusted for multiple comparisons using the Benjamini–Hochberg procedure.

Differential expression results as log2 fold change (log2FC) and adjusted *p*-values (adjP) for DESeq2 and Limma methods for the 19 signature genes are shown in ESM Table 9. *ARG2*, *PPP1R1A*, *CHL1*, *FBX032* and *SLCA2* are predicted to be significantly differentially expressed between glucose-treated and non-treated samples (adjusted p≤0.05) using DESeq2 and show a tendency for regulation with Limma, although for the latter method this is not statistically significant after correction for multiple comparisons. Confirmation with more samples will be required to better ascertain the precise regulation of these genes in islets upon glucose treatment.

Meta-analysis against pancreatic cancer cell signatures. To define whether our results were influenced by potential contamination of PPP islet samples with cancer cells, we used data from a recent study, which identified the transcriptome of four pancreatic cancer subtypes [24]. Comparing the differentially regulated genes from PPP islets to the signatures from each of the four cancer subtypes (as defined by statistical comparison of each subtype with the three others) revealed no enrichment of differentially regulated genes with ADEX, Immunogenic or Progenitor subtype signatures (ESM Table 15). A small but significant enrichment with the squamous signature was found, which however was also present for OD samples (OD p=4.75×10^{-6} for UP-regulated genes; PPP p=8.4×10^{-4} for UP-regulated genes; OD p=1.46×10^{-7} for DOWN-regulated genes; PPP p=3.32×10^{-5} for DOWN-regulated genes; ESM Table 9), suggesting that this signature, which contains 2,366 genes (ESM Table 9) and is the largest among the four cancer signatures, is more related to islet cells than to the other cancer subtypes. There are a few genes in the squamous cell signature that are differentially regulated in both OD and PPP. These genes are CAPN13, PCDH20, SCTR and TMEM37 (DOWN-regulated) and CD44 (UP-regulated). The fact that these genes are regulated in both OD and PPP suggests that they are more closely related to endocrine tissue rather than cancer tissue.

ESM Table 1 Primers used to prepare cDNA vectors

Gene	Accession no (NCBI)	Species	Forward primer	Reverse primer
ARG2	BC001350.1	Human	5′-ATGTCCCTAAGGGGCAGCC-3′	3′-GTTCGTGCACACTCTTAAATC-5′
PPP1R1A	NM_006741	Human	5′-ATGGAGCAAGACAACAGCCC-3′	3′-CCCTCGGTTGAGCCAGACT-5′
TMEM37	XM_005263597	Human	5′-ATGACCCGACCTGACTGTTC-3`	3′-GTGGGTAGGGACCCTTACT-5′
Tmem37	NM_019432	Mouse	5′-CACCATGACGGCCATCGGCGCGCAG-3`	3′-TATCAGAGAAGTCCCATCATAAC-5′

ESM Table 2 RT-qPCR primers

Gene	Accession no (NCBI)	Forward primer	Reverse primer
ACTB	NM_001101	5′-CATCGAGCACGGCATCGTCAC-3′	5′-CAGCACAGCCTGGATAGCAACG-3′
ANKRD23	NM_144994	5′-GTTGGTAAGTGGAGAAAGAGTTG-3′	5′-TCTTTGAACCAAGTTTTCCAAGTC-3′
ANKRD39	NM_016466	5′-ACGCTGGAGGAGATGGACTTC-3′	5′-CTTATGCAGACTGGTCATGCC-3′
ARG2	NM_001172	5′-CCTCCTGAACATTTTATTTTAAAG-3′	5′-GGATTGACTTCAACAAGATCCAG-3′
ASCL2	NM_005170	5′-TGCGCTGCAGCCGGCGGCGGCG-3′	5′-CGTGCGGCACGTGCTGCCGCAGCG-3′
CAPN13	NM_144575	5′-CTGGTGGACCTCACAGGAGG-3′	5′-CATCCAAAACTCGCCATCTTCC-3′
CD44	NM_000610	5′-CATGGACAAGTTTTGGTGGCAC-3′	5′-CCTTCTATGAACCCATACCTGC-3′
CHL1	NM_006614	5′-GTACATTAGTTAAAGTTACCTGGTC-3′	5′-GTGGCAGTGTCTTTATCAACTTTG-3′
FAM102B	NM_001010883	5′-TTTGCTGGATCAGGAAATACCAC-3′	5′-CTTTGCTGAAAGATCTGCTATTCC-3′
FBXO32	NM_058229	5′-TACAACTGAACATCATGCAGAGG-3′	5′-TACATCTTCTTCCAATCCAGCTG-3′
FFAR4	NM_181745	5′-AGCCTGGAGCGCATGGTGTG-3′	5′-GAGTAACTGATCACAATGACCAG-3′
G6PC2	NM_021176	5′-TTTTATGTCCAATGTTGGAGACC-3′	5′-TGGCCAGATGGACTTCCTGG-3′
HHATL	NM_020707	5′-GTGGTCTGTTCTTAACTGCTTC-3′	5′-GGAAGCCTGTAAGTATCAGGC-3′
HNF1A	NM_000545	5′-CACCAGAAAGCCGTGGTGGAG-3′	5′-GGAACAGGATCTGCTGGGATG-3′
KCNH8	NM_144633	5′-AATTCAAAGGAGAAATTATGTTCTAC-3′	5′-GTCTCCGGGCTGAGTCAAAGTG-3′
NSG1	NM_014392	5′-TCACCGAGAGGTTTAAG	5′-TCCCGGGCACTGGAGTCTT

		GTCTC-3´	G-3´
PCDH20	NM_022843	5´-TTTTCCTTAGACAGTGTCACAGG-3´	5´-GGACTGTTGTCATTTTTATCCAAC-3´
PDX1	NM_000209	5´-CAAAGCTCACGCGTGGAAAGG-3´	5´-GATGTGTCTCTCGGTCAAGTTC-3´
PPP1R1A	NM_006741	5´-CAATGTCTCCACGGCAACGGAAG-3´	5´-CTGTGTCTGGGATCCCAGGTG-3´
SCTR	NM_001980	5´-CAACTACATCCACATGCACCTG-3´	5´-AATGGCTGGAGAACCCCATCC-3´
SLC2A2	NM_000340	5´-TTCTGTCCAGAAAGCCCCAG-3´	5´-GCCCTGCCTTCTCCACAAG-3´
TMEM37	NM_183240	5´-TCCTTCATCCGGACCCTCATCATC-3´	5´-GAGGAAGGAGGCAGTGAATTC-3´
UNC5D	NM_080872	5´-CTTTTGTGAGGGAATGTCAGTG-3´	5´-TTGGGGTTTTATTTCATGAAGAGG-3´

ESM Table 3 Primers for *in situ* RT-PCR

Gene	Accession no (NCBI)	Forward primer	Reverse primer
ARG2	NM_001172	5′-TGGTTAGCAGAGCTGTGTCAGA-3′	5′-TTGGTCTTTGTCTCTTGCCAAT-3′
ASCL2	NM_005170	5′-GGTGAACTTGGGCTTCCAG-3′	5′-CCCTAACCAGCTGGAGAAGTC-3′
CHL1-iso1	NM_006614	5′-ATCTCCACTCAAGGCTGGTTT-3′	5′-TCCATTGCTTTCAACAGATCC-3′
CHL1-iso2	NM_001253387	5′-TGGTCAACAGTTCCAAAGGAC-3′	5′-CTGGGCTTTGATGGAGTTGTA-3′
CHL1-iso3	NM_001253388	5′-TCCATCGAACAATTCAGGAAC-3′	5′-TCATTGCGACTGTCCTTTTCT-3′
FAM102B	NM_001010883	5′-TACAAGCAAACTGTGTTCGCTG-3′	5′-TTCTAGAATGTCCACAGGCACC-3′
FFAR4	NM_181745.3	5′-AGGAAATTTCGATTTGCACACT-3′	5′-TCTTGCTTGAAGTTCTGGATCA-3′
HHATL	NM_020707	5′-TGATGTGGTTCACCTCCTTTC-3′	5′-CTTGAGCAGGTCAGCTAAGGA-3′
KCNH8	NM_144633	5′-GCCTATATTGCCGCTCTGTACT-3′	5′-CGGAGCACAGAAAGAGGTTTTG-3′
PPP1R1A	NM_006741	5′-TGACCAGTCATCCCCAGAGATA-3′	5′-GGAATCCAGTGGTGGTATATGG-3′
SCTR	NM_002980	5′-GGAAGGCCTCTACCTTCACAC-3′	5′-ACGATGTAGTGGATGCCAAAG-3′
SLC2A2	NM_000340	5′-GATCAATGCACCTCAACAGG-3′	5′-CCAATTTTGAAAACCCCATC-3′
TMEM37	NM_183240	5′-CGGTCCTTCTTTGAATCCTTC-3′	5′-ATTAGGGTGAAGCCGATGAGT-3′
UNC5D	NM_080872.2	5′-AGGGAATGTCAGTGCAGAAAAT-3′	5′-ACCTGTCAATGCAGAAGAGTCA-3′

ESM Table 4 Primers for chromatin immunoprecipitation

Gene	Ensemble ID	Forward primer	Reverse primer
ANKRD23/39	ENSG00000163126/ ENSG00000213337	5′-CAGCTGGATAGCAGGTCCCG-3′	5′-TCCTCCTGCAACACAGCCCA-3′
ARG2	ENSG00000081181	5′-CCAGCGCTCCCGTTATTCAGG-3′	5′-AATCTTCACGCCCGGCTGATG-3′
CAPN13	ENSG00000162949	5′-CTGTGGGCTCAGTGCAAGCACTG-3′	5′-GGGAAGATCACGAGAATCCTC-3′
CD44	ENSG00000026508	5′-CAGGTTCGGTCCGCCATCCT-3′	5′-CCCAGGCTGCGTGCCACCAA-3′
CHL1	ENSG00000134121	5′-CGAGGCTGTAAGGTCAATCTC-3′	5′-GCAAGTCCTCTTTGTTGG-3′
FAM102B	ENSG00000162636	5′-GGGCAACAGAGTAAGACTCTG -3′	5′-GCCTCAGTTGAATAGACCACCA-3′
FBXO32	ENSG00000156804	5′-ACCGCCAGTCCTGCCCGAGG-3′	5′-AGCATCCGCCCCGGGTGGCA-3′
G6PC2	NSG00000152254	5′-TTCAGCAGAGGAGGGCTGGT-3′	5′-AAGTGCTCTGATTCCCACCG-3′
KCNH8	ENSG00000183960	5′-ACGGAGAGGGAACAAAGGGG-3′	5′-GCTGAGCCTCCCGGTCTCCA-3′
PCDH20	ENSG00000197991	5′-GGGGCTGTACATGGAGTTCA-3′	5′-GGGGCTGAGGTTTACCACTTC-3′
PPP1R1A	ENSG00000135447	5′-AACCTTAACCCCGTCGTGGCT-3′	5′-AGCTGTCCACTACGACGGCT-3′
SCTR	ENSG00000080293	5′-CGTCAGATCCAGCAGTGAGT-3′	5′-GGCACCCAGCATTCCTGTTG-3′
SLC2A2	ENSG00000163581	5′-TGCTGATACCAGCCGTCTGA-3′	5′-GTTCTAGGGTGCATGCCGCT-3′
UNC5D	ENSG00000156687	5′-ACGTGGAGCGGCCTCTGGCT-3′	5′-GCCAATGAGCCGGGCTGGGG-3′

ESM Table 5 Determination of islet contamination

Annotation				Log intensities			
**Probe ID**	**Entrez ID**	**Symbol**	**Gene name**	**OD-ND**	**OD-T2D**	**PPP-ND**	**PPP-T2D**
209301_at	760	*CA2*	Carbonic anhydrase II	8.709	8.913	7.326	7.649
206208_at	762	*CA4*	Carbonic anhydrase IV	6.102	6.006	6.385	6.403
205043_at	1080	*CFTR*	Cystic fibrosis transmembrane conductance regulator (ATP-binding cassette sub-family C, member 7)	11.702	12.016	9.516	9.943
206297_at	11330	*CTRC*	Chymotrypsin C (caldecrin)	12.545	12.543	12.575	12.442
220275_at	50624	*CUZD1*	CUB and zona pellucida-like domains 1	12.655	12.828	11.526	11.734
214324_at	2813	*GP2*	Glycoprotein 2 (zymogen granule membrane)	11.657	11.741	12.346	12.220
200697_at	3098	*HK1*	Hexokinase 1	9.022	9.183	6.543	6.773
202934_at	3099	*HK2*	Hexokinase 2	9.339	9.560	5.850	6.483
201650_at	3880	*KRT19*	Keratin 19	12.606	12.749	8.176	8.488
208949_s_at	3958	*LGALS3*	Lectin, galactoside-binding, soluble, 3	12.036	12.156	8.908	9.590
213693_s_at	4582	*MUC1*	Mucin 1, cell surface associated	12.672	12.846	9.655	10.023
217109_at	4585	*MUC4*	Mucin 4, cell surface associated	6.160	6.820	absent	absent
205912_at	5406	*PNLIP*	Pancreatic lipase	13.594	13.533	13.440	13.197
206694_at	5407	*PNLIPRP1*	Pancreatic lipase-related protein 1	12.107	12.239	11.687	11.512
205869_at	5644	*PRSS1*	Protease, serine, 1 (trypsin 1)	12.915	12.929	12.585	12.346
211429_s_at	5265	*SERPINA1*	Serpin peptidase inhibitor, clade A (alpha-1 antiproteinase, antitrypsin), member 1	13.354	13.360	13.006	13.287
202936_s_at	6662	*SOX9*	SRY (sex determining region Y)-box 9	11.118	11.786	9.042	9.062
209875_s_at	6696	*SPP1*	Secreted phosphoprotein 1	12.732	13.023	9.627	10.759

OD, organ donors; ND, non-diabetic subjects; T2D, type 2 diabetes; PPP, phenotyped pancreatectomised patient.

ESM Table 6 Clinical characteristics of the global IMIDIA cohorts of organ donors (OD) and phenotyped pancreatectomized patients (PPP)

	Sex (F/M)	Age (years)	BMI (kg/m²)	Diabetes duration (years)	Blood glucose in ICU (mmol/l)	Fasting glucose (mmol/l)	HbA_{1c} (%) [mmol/mol]	Blood glucose at 2 h in the OGTT (mmol/l)	Histopathology		
									Chronic pancreatitis	Benign tumour	Malign tumour
**OD cohort (n=243)**											
**ND 204** (84.0%)	108/96	61±17	25.6±3.8 (n=196)	–	8.5±2.2 (n=178)	–	–	–	–	–	–
**T2D 39** (16.0%)	12/27	73±8	26.2±3.7 (n=39)	10.4±6.9 (n=32)	11.8±3.7 (n=37)	–	–	–	–	–	–
**PPP cohort (n=201)**											
**ND 70** (34.8%)	35/35	61±13	24.5±4.0	–	–	5.3±0.6 (n=60)	5.5±0.5 [37±5.5] (n=69)	5.9±1.3 (n=53)	11 (16%)	22 (31%)	37 (53%)
**T2D 54** (26.9%)	20/34	65±13*	25.6±5.3	10.7±8.9	–	8.2±2.4*** (n=47)	7.4±1.2*** [57±13.1] (n=52)	–	14 (26%)	6 (11%)	34 (63%)
**IGT 30** (14.9%)	13/17	63±12	26.1±4.1	–	–	5.5±0.6 (n=30)	5.6±0.4 [38±4.4] (n=30)	9.3±0.8*** (n=30)	4 (13%)	10 (33%)	16 (54%)
**T3cD 46** (22.9%)	18/28	67±10**	25.9±4.2	0.05±0.1	–	7.3±4.3** (n=44)	6.6±1.4*** [49±15.3] (n=46)	12.8±2.7*** (n=23)	9 (19%)	5 (11%)	32 (70%)
**T1D 1** (0.5%)	0/1	53	26.9	6.0	–	7.6	7.8	–	–	–	1 (100%)

F, female; M, male; ICU, intensive care unit; IGT, impaired glucose tolerant subject; ND, non-diabetic subject; T1D, type 1 diabetic subject; T2D, type 2 diabetic subject; T3cD, type 3c diabetic subject. Except for sex, the values are means ± standard deviation (two-tailed *t*-test; *p<0.05, **p<0.01 and ***p<0.001) versus ND.

ESM Table 7 Differentially regulated genes in islets from OD and PPP.

See Excel spreadsheet (provided as a separate file)

Related to Fig. 2 and Table 2. Adj. *p*, *p*-value adjusted for multiple hypothesis tests using the Benjamini–Hochberg method; OD, organ donor; PPP, partially pancreatectomized patient; probe ID, probe set ID; reg. OD, regulated in organ donor; reg. PPP, regulated in phenotyped pancreatectomized patient.

ESM Table 8 Top-20 regulated genes (absolute fold-change of ≥1.5) in islets of T2D OD versus islets of ND OD and in islets of T2D PPP versus islets of ND PPP

**OD islets**						
**Entrez ID**	**Symbol**	**Gene name**	**Probe ID**	**Ratio**	***p***	**Adj. *p***
10058	*ABCB6*	ATP-binding cassette, sub-family B (MDR/TAP), member 6	203192_at	0.645	$3.44×10^{−12}$	$1.020×10^{−7}$
57467	*HHATL*	Hedgehog acyltransferase-like	223572_at	0.388	$1.86×10^{−8}$	$1.098×10^{−4}$
6514	*SLC2A2*	Solute carrier family 2 (facilitated glucose transporter), member 2	206535_at	0.273	$3.93×10^{−8}$	$1.657×10^{−4}$
8544	*PIR*	Pirin (iron-binding nuclear protein)	207469_s_at	0.586	$1.00×10^{−7}$	$3.073×10^{−4}$
5502	*PPP1R1A*	Protein phosphatase 1, regulatory (inhibitor) subunit 1A	235129_at	0.419	$1.25×10^{−7}$	$3.073×10^{−4}$
384	*ARG2*	Arginase 2	203946_s_at	0.605	$2.47×10^{−7}$	$3.286×10^{−4}$
56605	*ERO1LB*	ERO1-like beta (S. cerevisiae)	220012_at	0.554	$3.08×10^{−7}$	$3.627×10^{−4}$
64130	*LIN7B*	Lin-7 homolog B (C. elegans)	219760_at	0.589	$4.08×10^{−7}$	$4.155×10^{−4}$
7057	*THBS1*	Thrombospondin 1	201110_s_at	1.761	$4.59×10^{−7}$	$4.515×10^{−4}$
3375	*IAPP*	Islet amyloid polypeptide	207062_at	0.580	$6.12×10^{−7}$	$5.312×10^{−4}$
79623	*GALNT14*	UDP-N-acetyl-alpha-D-galactosamine:polypeptide N-acetylgalactosaminyltransferase 14 (GalNAc-T14)	219271_at	0.493	$1.08×10^{−6}$	$7.407×10^{−4}$
773 /// 100507353	*CACNA1A /// LOC100507353*	Calcium channel, voltage-dependent, P/Q type, alpha 1A subunit /// uncharacterized LOC100507353	1558945_s_at	0.469	$1.39×10^{−6}$	$7.641×10^{−4}$
486 /// 100533181	*FXYD2 /// FXYD6-FXYD2*	FXYD domain containing ion transport regulator 2 /// FXYD6-FXYD2 readthrough	207434_s_at	0.594	$1.54×10^{−6}$	$7.641×10^{−4}$
2740	*GLP1R*	Glucagon-like peptide 1 receptor	208400_at	0.526	$1.38×10^{−6}$	$7.641×10^{−4}$
89765	*RSPH1*	Radial spoke head 1 homolog (Chlamydomonas)	230093_at	0.654	$1.55×10^{−6}$	$7.641×10^{−4}$
4093	*SMAD9*	SMAD family member 9	227719_at	0.554	$1.58×10^{−6}$	$7.641×10^{−4}$
84623	*KIRREL3*	Kin of IRRE like 3 (Drosophila)	240402_at	0.445	$2.14×10^{−6}$	$8.332×10^{−4}$
3428	*IFI16*	Interferon, gamma-inducible protein 16	208965_s_at	1.525	$2.26×10^{−6}$	$8.663×10^{−4}$
5798	*PTPRN*	Protein tyrosine phosphatase, receptor type, N	204945_at	0.548	$2.73×10^{−6}$	$9.673×10^{−4}$
309	*ANXA6*	Annexin A6	200982_s_at	0.654	$3.02×10^{−6}$	$1.002×10^{−3}$

PPP islets						
**Entrez ID**	**Symbol**	**Gene name**	**Probe ID**	**Ratio**	***p***	**Adj *p***
26577	*PCOLCE2*	Procollagen C-endopeptidase enhancer 2	219295_s_at	6.519	1.23×10^{-15}	3.65×10^{-11}
392617	*ELFN1*	Extracellular leucine-rich repeat and fibronectin type III domain containing 1	229581_at	4.391	7.10×10^{-15}	1.05×10^{-10}
229	*ALDOB*	Aldolase B, fructose-bisphosphate	204705_x_at	4.588	1.85×10^{-13}	1.24×10^{-9}
384	*ARG2*	Arginase 2	203946_s_at	0.463	2.98×10^{-13}	1.47×10^{-9}
140738	*TMEM37*	Transmembrane protein 37	227190_at	0.504	9.09×10^{-13}	3.84×10^{-9}
430	*ASCL2*	Achaete-scute complex homolog 2 (Drosophila)	229215_at	0.463	5.97×10^{-12}	1.74×10^{-8}
145270	*PRIMA1*	Proline rich membrane anchor 1	230087_at	4.975	7.65×10^{-12}	1.74×10^{-8}
6514	*SLC2A2*	Solute carrier family 2 (facilitated glucose transporter), member 2	206535_at	0.362	7.52×10^{-12}	1.74×10^{-8}
27122	*DKK3*	Dickkopf WNT signaling pathway inhibitor 3	221127_s_at	2.335	1.35×10^{-11}	2.86×10^{-8}
23017	*FAIM2*	Fas apoptotic inhibitory molecule 2	203619_s_at	3.826	1.47×10^{-11}	2.91×10^{-8}
151647	*FAM19A4*	Family with sequence similarity 19 (chemokine (C-C motif)-like), member A4	242348_at	0.379	1.47×10^{-10}	2.41×10^{-7}
5172	*SLC26A4*	Solute carrier family 26, member 4	206529_x_at	3.379	2.11×10^{-10}	3.29×10^{-7}
57393	*TMEM27*	Transmembrane protein 27	223784_at	0.572	4.25×10^{-10}	6.29×10^{-7}
2906	*GRIN2D*	Glutamate receptor, ionotropic, N-methyl D-aspartate 2D	229883_at	0.423	1.03×10^{-9}	1.32×10^{-6}
203111	*C8orf47*	Chromosome 8 open reading frame 47	1552389_at	0.554	3.61×10^{-9}	3.58×10^{-6}
5502	*PPP1R1A*	Protein phosphatase 1, regulatory (inhibitor) subunit 1A	235129_at	0.475	5.80×10^{-9}	5.54×10^{-6}
192668	*CYS1*	Cystin 1	228739_at	0.395	6.92×10^{-9}	6.29×10^{-6}
170691	*ADAMTS17*	ADAM metallopeptidase with thrombospondin type 1 motif, 17	1552727_s_at	3.053	1.68×10^{-8}	1.42×10^{-5}
10752	*CHL1*	Cell adhesion molecule with homology to L1CAM (close homolog of L1)	204591_at	0.446	1.95×10^{-8}	1.56×10^{-5}
83876	*MRO*	Maestro	231358_at	0.432	2.08×10^{-8}	1.62×10^{-5}

Related to Fig. 2 and Table 2. Adj. *p*, *p*-value adjusted for multiple hypothesis tests using the Benjamini–Hochberg method; ND, non-diabetic subject; OD, organ donor; PPP, phenotyped pancreatectomized patient; probe ID, probe set ID; T2D, type 2 diabetic subject.

ESM Table 9 RNAseq differential expression of the 19 signature genes identified by microarrays

Gene symbol	DESeq2 log2FC	DESeq2 adj. *p*	Limma log2FC	Limma adj. *p*
ARG2	0.8662	2.32×10^{-6}	0.8906	0.115909107
PPP1R1A	0.7243	9.03×10^{-6}	0.7042	0.094173095
CHL1	−0.8072	0.019487108	−0.8088	0.250944235
FBXO32	−0.5505	0.026054668	−0.5641	0.222276879
SLC2A2	0.5459	0.035281768	0.5444	0.329481919
NSG1	−0.4456	0.43006851	−0.5198	0.454843133
FAM102B	−0.5884	0.58224798	−0.5316	0.599328157
UNC5D	0.4089	0.771939204	0.1052	0.980593289
G6PC2	0.1976	0.80802655	0.2163	0.697640805
ANKRD39[1]	0.2133	0.863044479	0.1699	0.802122995
TMEM37	−0.1522	0.953608152	−0.1865	0.715048321
CD44	−0.1088	0.973151446	−0.1373	0.757562348
HHATL	0.2231	0.986919081	0.2694	0.859040284
SCTR	0.1865	0.986919081	0.1870	0.879517345
CAPN13	−0.0428	0.999689273	0.0013	0.999544913
FFAR4	0.0945	0.999689273	0.1962	0.946451699
ANKRD23[1]	−0.0542	1	0.0696	0.9846822
ASCL2	0.7319	1	0.4520	0.929239565
KCNH8	−0.2335	1	0.0023	0.999544913
PCDH20	0.1503	1	−0.5325	0.334922553

DE, differential expression; log2FC, log2 fold change; adj. *p*, adjusted *p*-value.

[1]*ANKRD39* and *ANKRD23* were not distinguishable by microarray.

ESM Table 10 Top-2ß regulated GOs for T2D vs. ND regulated probesets.

GOBPID	Term	*P*-value	Count	Size
**Top-20 GOs for T2D vs ND downregulated probe sets in OD islets**				
GO:0007267	Cell-cell signaling	4.091×10^{-25}	69	771
GO:0023061	Signal release	6.532×10^{-24}	44	308
GO:0030072	Peptide hormone secretion	4.371×10^{-19}	31	185
GO:0046879	Hormone secretion	7.427×10^{-19}	33	218
GO:0009914	Hormone transport	1.143×10^{-18}	33	221
GO:0002790	Peptide secretion	1.147×10^{-18}	31	191
GO:0051046	Regulation of secretion	4.334×10^{-18}	44	430
GO:0010817	Regulation of hormone levels	5.722×10^{-18}	38	318
GO:1903530	Regulation of secretion by cell	8.089×10^{-18}	42	397
GO:0030073	Insulin secretion	1.504×10^{-17}	28	164
GO:0007268	Synaptic transmission	2.265×10^{-17}	44	449
GO:0015833	Peptide transport	3.849×10^{-17}	32	231
GO:0046883	Regulation of hormone secretion	1.626×10^{-16}	28	179
GO:0042886	Amide transport	1.742×10^{-16}	32	243
GO:0090276	Regulation of peptide hormone secretion	1.844×10^{-16}	26	151
GO:0002791	Regulation of peptide secretion	3.588×10^{-16}	26	155
GO:0050796	Regulation of insulin secretion	2.644×10^{-15}	24	139
GO:0097479	Synaptic vesicle localization	5.884×10^{-15}	21	104
GO:0032940	Secretion by cell	7.003×10^{-15}	49	642
GO:0071705	Nitrogen compound transport	1.701×10^{-14}	44	537
**Top-20 GOs for T2D vs ND downregulated probe sets in PPP islets**				
**GOBPID**	**Term**	***P*-value**	**Count**	**Size**
GO:0043583	Ear development	1.498×10^{-5}	6	139
GO:0010817	Regulation of hormone levels	2.255×10^{-5}	8	311
GO:0046879	Hormone secretion	1.669×10^{-4}	6	214
GO:0009914	Hormone transport	1.800×10^{-4}	6	217
GO:0048729	Tissue morphogenesis	1.897×10^{-4}	8	421
GO:0050796	Regulation of insulin secretion	2.362×10^{-4}	5	145
GO:0035295	Tube development	3.170×10^{-4}	8	454
GO:0090276	Regulation of peptide hormone secretion	3.217×10^{-4}	5	155
GO:0002009	Morphogenesis of an epithelium	3.259×10^{-4}	7	343
GO:0009790	Embryo development	3.325×10^{-4}	10	714
GO:0002791	Regulation of peptide secretion	3.619×10^{-4}	5	159
GO:0050708	Regulation of protein secretion	4.291×10^{-4}	6	255
GO:0009887	Organ morphogenesis	4.650×10^{-4}	9	608
GO:0030073	Insulin secretion	4.788×10^{-4}	5	169
GO:0046883	Regulation of hormone secretion	5.614×10^{-4}	5	175
GO:0044707	Single-multicellular organism process	5.759×10^{-4}	27	4085
GO:0010721	Negative regulation of cell development	7.050×10^{-4}	5	184
GO:0048562	Embryonic organ morphogenesis	7.403×10^{-4}	5	186
GO:0090087	Regulation of peptide transport	7.403×10^{-4}	5	186
GO:0030072	Peptide hormone secretion	7.585×10^{-4}	5	187

Related to Fig. 4. OD, organ donor; PPP, phenotyped pancreatectomized patient; T2D, type 2 diabetic subject.

ESM Table 11 Downregulated genes assigned to the insulin secretion Gene Ontology (GO: 0030073) for islets from T2D OD and T2D PPP

T2D vs ND downregulated genes in OD islets assigned to 'insulin secretion' GO			
**Probe ID**	**Symbol**	**Ratio**	**Adj. *p***
210246_s_at	*ABCC8*	0.509	1.223×10^{-3}
1552519_at	*ACVR1C*	0.649	4.461×10^{-2}
209869_at	*ADRA2A*	0.646	2.229×10^{-2}
1558944_at	*CACNA1A*	0.528	1.037×10^{-3}
1558945_s_at	*CACNA1A*	0.469	7.641×10^{-4}
204811_s_at	*CACNA2D2*	0.626	3.070×10^{-3}
223500_at	*CPLX1*	0.635	1.003×10^{-2}
205630_at	*CRH*	0.463	8.142×10^{-3}
1561507_at	*FFAR1*	0.579	7.271×10^{-3}
221453_at	*G6PC2*	0.616	3.440×10^{-2}
231291_at	*GIPR*	0.664	1.557×10^{-2}
208400_at	*GLP1R*	0.526	7.641×10^{-4}
208401_s_at	*GLP1R*	0.602	1.002×10^{-3}
217057_s_at	*GNAS*	0.528	7.948×10^{-3}
229380_at	*ILDR2*	0.573	2.654×10^{-3}
206762_at	*KCNA5*	0.655	3.043×10^{-2}
216096_s_at	*NRXN1*	0.632	7.760×10^{-3}
205825_at	*PCSK1*	0.644	7.105×10^{-3}
1554789_a_at	*PDE8B*	0.566	6.236×10^{-3}
204945_at	*PTPRN*	0.548	9.673×10^{-4}
219140_s_at	*RBP4*	0.577	5.728×10^{-3}
222049_s_at	*RBP4*	0.564	6.530×10^{-3}
1552673_at	*RFX6*	0.620	8.586×10^{-3}
229823_at	*RIMS2*	0.657	1.458×10^{-2}
206535_at	*SLC2A2*	0.273	1.657×10^{-4}
1552985_at	*SLC30A8*	0.654	3.325×10^{-2}
202508_s_at	*SNAP25*	0.634	1.026×10^{-2}
204729_s_at	*STX1A*	0.549	2.029×10^{-3}
202260_s_at	*STXBP1*	0.658	1.559×10^{-3}
240236_at	*STXBP5L*	0.583	5.083×10^{-3}
232025_at	*SYT7*	0.629	2.233×10^{-3}
**T2D vs ND downregulated genes in PPP islets assigned to 'insulin secretion' GO**			
**Probe ID**	**Symbol**	**Ratio**	**Adj. *p***
221453_at	*G6PC2*	0.643	2.773×10^{-2}
210938_at	*PDX1*	0.631	6.419×10^{-5}
209992_at	*PFKFB2*	0.644	2.833×10^{-2}
206535_at	*SLC2A2*	0.362	1.743×10^{-8}
223784_at	*TMEM27*	0.572	6.288×10^{-7}

Related to Fig. 4. OD, organ donor; Adj. *p*, *p*-value adjusted for multiple hypothesis tests using the Benjamini–Hochberg method; PPP, phenotyped pancreatectomized patient; T2D, type 2 diabetic subject.

ESM Table 12 Downstream functions predicted to be significantly increased or decreased among the differentially regulated probe sets in islets from T2D OD and T2D PPP

**Ingenuity downstream functions for T2D vs ND regulated probe sets in OD islets**				
**Diseases or functions annotation**	**Predicted activation state**	**Activation z-score**	***p***	**Gene number**
Coordination	Decreased	−3.225	2.03×10^{-5}	18
Size of body	Decreased	−3.220	3.35×10^{-6}	52
Transport of metal ion	Decreased	−2.822	2.98×10^{-7}	26
Long-term potentiation	Decreased	−2.796	5.30×10^{-4}	17
Transport of metal	Decreased	−2.703	5.86×10^{-9}	30
Transport of monovalent inorganic cation	Decreased	−2.646	1.79×10^{-4}	16
Conditioning	Decreased	−2.407	1.51×10^{-5}	18
Concentration of cyclic AMP	Decreased	−2.380	1.09×10^{-6}	19
Release of neurotransmitter	Decreased	−2.325	2.80×10^{-12}	24
Transport of cation	Decreased	−2.313	1.53×10^{-5}	27
Neoplasia of epithelial tissue	Decreased	−2.296	2.91×10^{-4}	329
Rearing	Decreased	−2.282	4.09×10^{-5}	11
Growth of neurites	Decreased	−2.211	7.15×10^{-7}	35
Outgrowth of neurites	Decreased	−2.207	1.89×10^{-6}	31
Quantity of vesicles	Decreased	−2.170	5.37×10^{-4}	9
Accumulation of cyclic AMP	Decreased	−2.115	2.16×10^{-4}	13
Differentiation of tumour cell lines	Decreased	−2.105	1.61×10^{-3}	22
Outgrowth of cells	Decreased	−2.078	3.20×10^{-6}	32
Heart septal defect	Decreased	−2.033	1.88×10^{-3}	9
Synaptic transmission	Decreased	−2.018	1.83×10^{-8}	31
Neonatal death	Increased	2.121	4.50×10^{-7}	34
Hyperesthesia	Increased	2.157	1.66×10^{-3}	9
Ataxia	Increased	2.161	1.03×10^{-3}	17
Organismal death	Increased	2.246	1.30×10^{-6}	116
Seizure disorder	Increased	2.361	2.63×10^{-9}	40
Seizures	Increased	2.361	1.53×10^{-8}	35
Movement disorders	Increased	2.545	2.32×10^{-7}	65
Proliferation of tumour cell lines	Increased	2.760	3.50×10^{-4}	69
Hyperactive behaviour	Increased	2.920	3.82×10^{-6}	15

**Ingenuity downstream functions for T2D vs ND regulated probe sets in PPP islets**				
**Diseases or functions annotation**	**Predicted activation state**	**Activation z-score**	***p***	**Gene number**
Quantity of beta islet cells	Decreased	−2.805	1.43×10^{-4}	8
Quantity of secretory structure	Decreased	−2.503	2.27×10^{-2}	12
Cell survival	Decreased	−2.460	1.07×10^{-2}	84
Quantity of islet cells	Decreased	−2.447	1.06×10^{-3}	9
Quantity of apud cells	Decreased	−2.442	1.63×10^{-4}	9
Clathrin mediated endocytosis	Decreased	−2.407	2.11×10^{-2}	6
Cell viability	Decreased	−2.340	1.66×10^{-2}	77
Quantity of neuroendocrine cells	Decreased	−2.299	8.50×10^{-4}	11
Size of brain	Decreased	−2.164	2.57×10^{-2}	12
Quantity of protein in blood	Decreased	−2.076	4.95×10^{-4}	40
Phagocytosis of phagocytes	Increased	2.011	1.15×10^{-2}	11
Phagocytosis of myeloid cells	Increased	2.154	4.86×10^{-3}	12
Phagocytosis of neutrophils	Increased	2.190	6.38×10^{-3}	6
Quantity of carbohydrate	Increased	2.342	7.61×10^{-3}	36
Seizure disorder	Increased	2.433	2.69×10^{-3}	35
Organismal death	Increased	2.453	1.45×10^{-3}	146

Related to Fig. 2 and Fig. 4. OD, organ donor; PPP, phenotyped pancreatectomized patient; T2D, type 2 diabetic subject.

ESM Table 13 Upstream regulators predicted to be significantly activated or inhibited among the differentially regulated probe sets in islets from OD and PPP

Upstream regulator	Log ratio	Molecule type	Predicted activation state	Activation z-score	*P*-value
**Ingenuity upstream regulators of T2D vs ND regulated probe sets in OD islets**					
TNF	0.515	Cytokine	Activated	4.078	1.49×10^{-5}
REST	0.559	Transcription regulator	Activated	3.395	2.92×10^{-18}
IL1B	0.817	Cytokine	Activated	3.031	7.95×10^{-8}
STAT1	0.086	Transcription regulator	Activated	2.630	2.24×10^{-2}
IL1A	0.486	Cytokine	Activated	2.595	5.67×10^{-3}
IFI16	0.613	Transcription regulator	Activated	2.412	5.81×10^{-3}
AR	−0.215	Ligand-dependent nuclear receptor	Activated	2.377	2.33×10^{-2}
FOXO1	0.111	Transcription regulator	Activated	2.270	6.61×10^{-3}
TLR3	0.446	Transmembrane receptor	Activated	2.206	4.90×10^{-2}
PRKCD	−0.096	Kinase	Activated	2.166	3.78×10^{-2}
MAP3K8	0.292	Kinase	Activated	2.159	1.12×10^{-2}
IKBKB	0.079	Kinase	Activated	2.126	8.36×10^{-4}
CTNNB1	0.106	Transcription regulator	Activated	2.064	6.04×10^{-4}
NTRK2	−0.136	Kinase	Inhibited	−2.000	3.60×10^{-2}
TAB1	−0.089	Enzyme	Inhibited	−2.000	3.24×10^{-3}
PAX6	−0.596	Transcription regulator	Inhibited	−2.070	2.30×10^{-4}
BDNF	0.027	Growth factor	Inhibited	−2.166	2.37×10^{-7}
NEUROD1	–0.545	Transcription regulator	Inhibited	−2.619	1.10×10^{-5}
ADCYAP1	–0.811	Other	Inhibited	−2.720	1.34×10^{-2}
**Ingenuity upstream regulators of T2D vs ND regulated probe sets in PPP islets**					
**Upstream regulator**	**Log ratio**	**Molecule type**	**Predicted activation state**	**Activation z-score**	***P*-value**
RICTOR	0.130	Other	Activated	2.848	1.40×10^{-2}
SRC	–0.052	Kinase	Inhibited	−2.156	2.13×10^{-3}
HNF1A	–0.328	Transcription regulator	Inhibited	−2.573	8.81×10^{-4}

Related to Fig. 2 and Fig. 5. OD, organ donor; PPP, phenotyped pancreatectomized patient.

ESM Table 14 Islet signature genes with predicted binding sites for HNF1A and PDX1. Number of predicted binding sites in the promoters is indicated.

**Gene**	**ENTREZ ID**	**HNF1A**	**PDX1**
ANKRD23/39	51239/200539		1
ARG2	384		5
ASCL2	430		
CAPN13	92291		13
CHL1	10752		39
FFAR4	338557		
G6PC2	E57818		15
HHATL	57467		1
NSG1/D4S234E	27065		
PCDH20	64881		2
PPP1R1A	5502		3
SCTR	6344		1
SLC2A2	6514		5
TMEM37	140738		
UNC5D	137970	1	33
CD44	960	2	19
FAM102B	284611	1	2
FBXO32	114907		19
KCNH8	131096		15

ESM Table 15 Bailey Comparison

See Excel spreadsheet (provided as a separate file).

Related to Fig. 2 and Table 2. Differential expression analysis results for ADEX, Immunogenic, Progenitor and Squamous pancreatic cancer subtypes were downloaded from Bailey et al. [7]. The comparisons of each of the four subtypes against the other three subtypes were used to define a specific gene expression signature for each subtype, which were then compared to the differential expression results from PPP and OD samples (T2D vs ND). Only genes with a fold-change ≥1.5 and adjusted *p*-value ≤0.05 were considered to be differentially expressed for each comparison. The number of upregulated or downregulated genes, as well as the total number of DE genes in OD and PPP T2D vs ND that overlap with each signature is shown. Significance of overlap between PPP or OD T2D vs ND comparisons and each of the pancreatic cancer subtype signatures was calculated using the hypergeometric distribution with the background as the total number of expressed genes in the OD or PPP samples. The genes overlapping between OD/PPP and the tumour signatures are also shown.

ESM Table 16 Islet characteristics

	Mean no of LCM islets/patient	Mean LCM islet volume [µm³]/patient	Islet RNA/patient (ng) [range]	RIN
ND 32	42±15	29,673,810±11,210,130	4.9–207.8	6.6±0.7
T2D 36	47±14	32,597,030±11,405,690	5.9–784.0	6.0±0.8
IGT 15	39±13	25,991,280±6,031,880	4.8–335.0	6.3±0.7
T3cD 20	45±11	28,103,890±6,420,620	7.5–163.3	6.4±0.7
Total 103	44±14	29,854,390±10,048,130	4.8–784.0	6.3±0.8

LCM, laser capture microdissection; RIN, RNA Integrity Number; ND, non-diabetic subjects; T2D, type 2 diabetes; IGT, impaired glucose tolerance; T3cD, type 3c diabetes.

ESM Table 17 Clinical and functional traits used for the module correlations

**Trait**	**Sample type**
Sex (M/F)	OD+PPP
Age (years)	OD+PPP
Height (cm)	OD+PPP
Weight (kg)	OD+PPP
BMI (kg/m^2)	OD+PPP
Diabetes status (T2D, T3D, IGT, ND)	OD+PPP
Blood glucose in ICU (mmol/l)	OD
Fructosamine (μmol/l)	OD
Glucose-induced ISI	OD
Glibenclamide-induced ISI	OD
Arginine-stimulated insulin secretion index	OD
Basal insulin secretion at 3.3 mmol/l glucose (pg/islet/min)	OD
Glucose-stimulated insulin secretion at 16.7 mmol/l glucose (pg/islet/min)	OD
Glibenclamide-stimulated insulin secretion at 100 mmol/l glibenclamide (pg/islet/min)	OD
Arginine-stimulated insulin secretion at 100 mm arginine (pg/islet/min)	OD
Oral glucose tolerance (AUC)	PPP
Blood glucose i.vi OGTT at 0 h (mmol/l)	PPP
Blood glucose i.v. OGTT at 1 h (mmol/l)	PPP
Blood glucose i.v. OGTT at 2 h (mmol/l)	PPP
HbA_{1c} (%) [mmol/mol]	PPP
HOMA1 IR Index	PPP
HOMA1 B Index	PPP
HOMA1 S Index	PPP
Fasting insulin i.p. (pmol/l)	PPP
Fasting glucose i.p. (mmol/l)	PPP
Fasting insulin i.p. (nmol/l)	PPP
Insulin IS 1 h (nmol/l)	PPP
Insulin IS 2 h (nmol/l)	PPP
Fasting C-peptide IS (nmol/l)	PPP
C-peptide IS 1 h (nmol/l)	PPP
C-peptide IS 2 h (nmol/l)	PPP
Fasting pro-insulin IS (pmol/l)	PPP
Pro-insulin IS 1 h (pmol/l)	PPP
Pro-insulin IS 2 h (pmol/l)	PPP
K IS (mmol/l)	PPP
Mg IS (mmol/l)	PPP
C-peptide IS 1 h/fasting C peptide (ratio)	PPP
Insulin IS 1 h/fasting insulin IS (ratio)	PPP

Pro-insulin IS 1 h/fasting pro-insulin IS (ratio)	PPP

M, male; F, female; OD, organ donor; PPP, phenotyped pancreatectomised patient; T2D, type 2 diabetes; T3cD, type 3c diabetes; IGT, impaired glucose tolerance; ND, non-diabetic subjects; ICU, intensive care unit; OGTT, oral glucose tolerance test; ISI, insulin stimulation index; IS, in serum; AUC, area under the concentration–time curve.

ESM Table 18 Significance of selected OD and PPP modules

**Module**	**Type**	**Z-score**	***p***
Blue	OD	80.54	<0.001
Skyblue	OD	73.89	<0.001
Darkviolet	OD	47.22	<0.001
Plum1	OD	35.99	<0.001
Antiquewhite4	PPP	80.11	<0.001
Firebrick	PPP	86.72	<0.001
Deeppink2	PPP	68.73	<0.001
Lightpink4	PPP	76.46	<0.001
Coral3	PPP	23.91	<0.001

OD, organ donor; PPP, phenotyped pancreatectomised patient.

ESM Table 19 Modules and correlated traits used for the sequenced-based TF network generation

Module	Sample source	Correlated trait
Antiquewhite4	PPP	Fasting insulin
Blue	OD	Insulin stimulation index
Darkviolet	OD	Insulin stimulation index
Deeppink2	PPP	Mg
Lightpink4	PPP	Blood glucose at 2 h
Plum1	OD	Glibenclamide stimulation index
Skyblue	OD	Glucose stimulation index

OD, organ donor; PPP, phenotyped pancreatectomised patient; Mg, Magnesium.

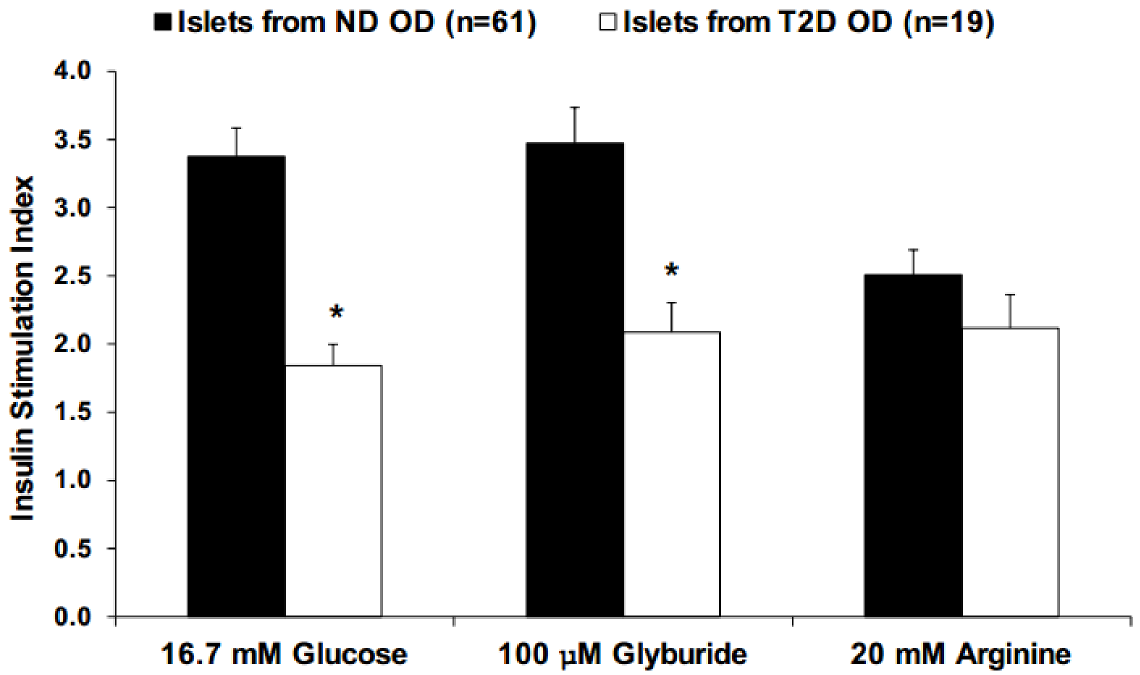

ESM Fig. 1 T2D OD islets secrete less insulin in response to glucose or glyburide. Related to Table 1. Hand-picked islets of ND and T2D OD were exposed to 3.3 mmol/l glucose for 45 min to assess basal insulin release (pmol/10 islets/h, mean±SE) and then challenged with 16.7 mmol/l glucose, 3.3 mmol/l glucose plus 100 µmol/l glyburide, or 3.3 mmol/l glucose plus 20 mmol/l arginine. Basal insulin release was 0.133±0.008 and 0.129±0.004 for T2D and ND OD islets, respectively (p=0.77, two-tailed Student's t-test). Insulin secretion in response to 16.7 mmol/l glucose (0.258±0.037vs 0.491±0.050, p=0.01) and glyburide (0.283±0.033 vs 0.457±0.037, p=0.01), but not to arginine (0.283±0.033 vs 0.312±0.025, p=0.47), was lower in T2D islets. Accordingly, the insulin stimulation index (stimulated over basal insulin release, ISI) was lower in T2D islets after stimulation with glucose or glyburide (*p<0.01, two-tailed Student's t-test).
ND, non-diabetic; T2D, type 2 diabetes

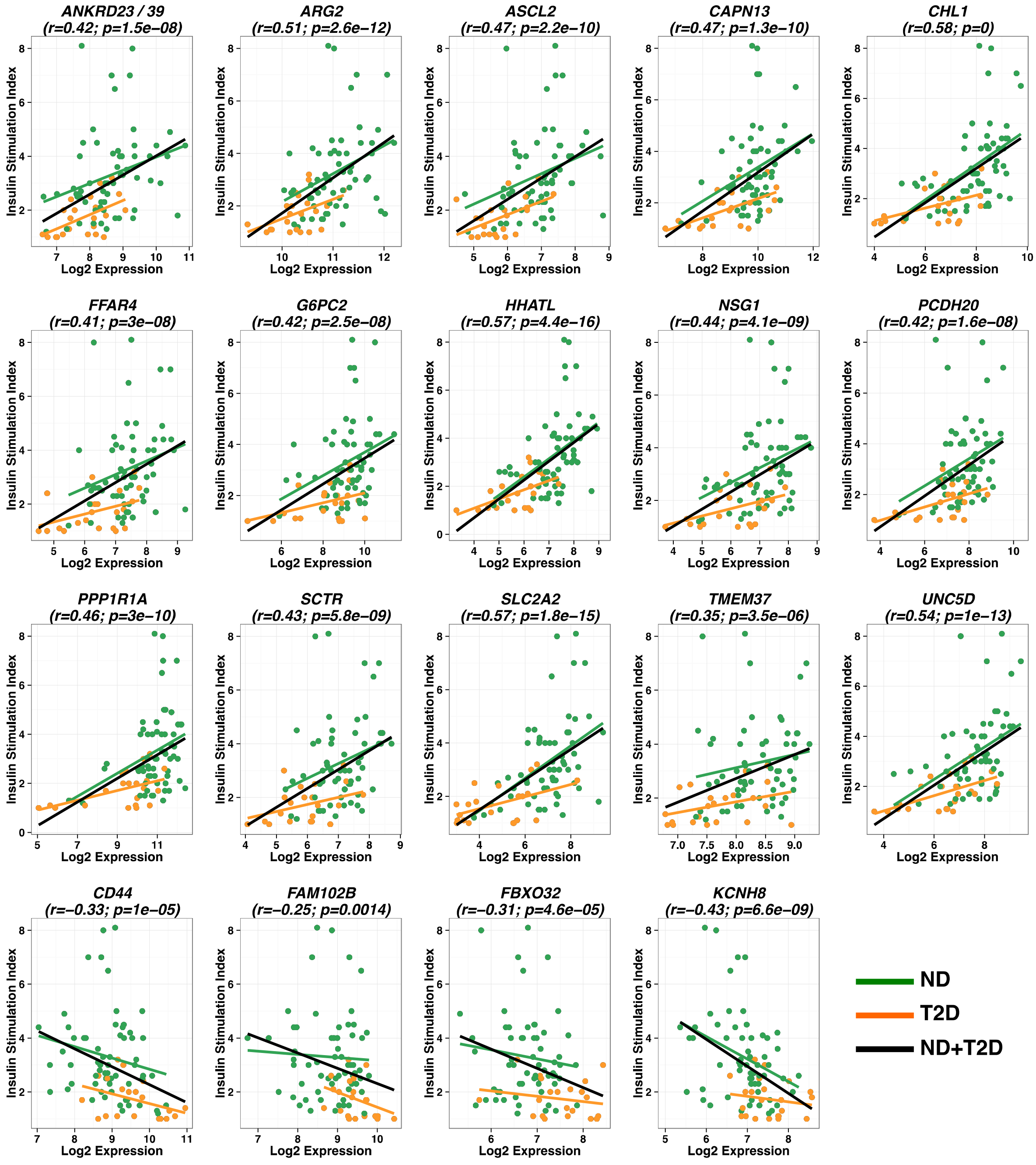

[illegible] re [illegible] es [illegible] A regression line for the ND and T2D samples combined is coloured black. Pearson's correlation coefficients (r) and the corresponding p-values are indicated at the top of each plot.
ND, non-diabetic; T2D, type 2 diabetes

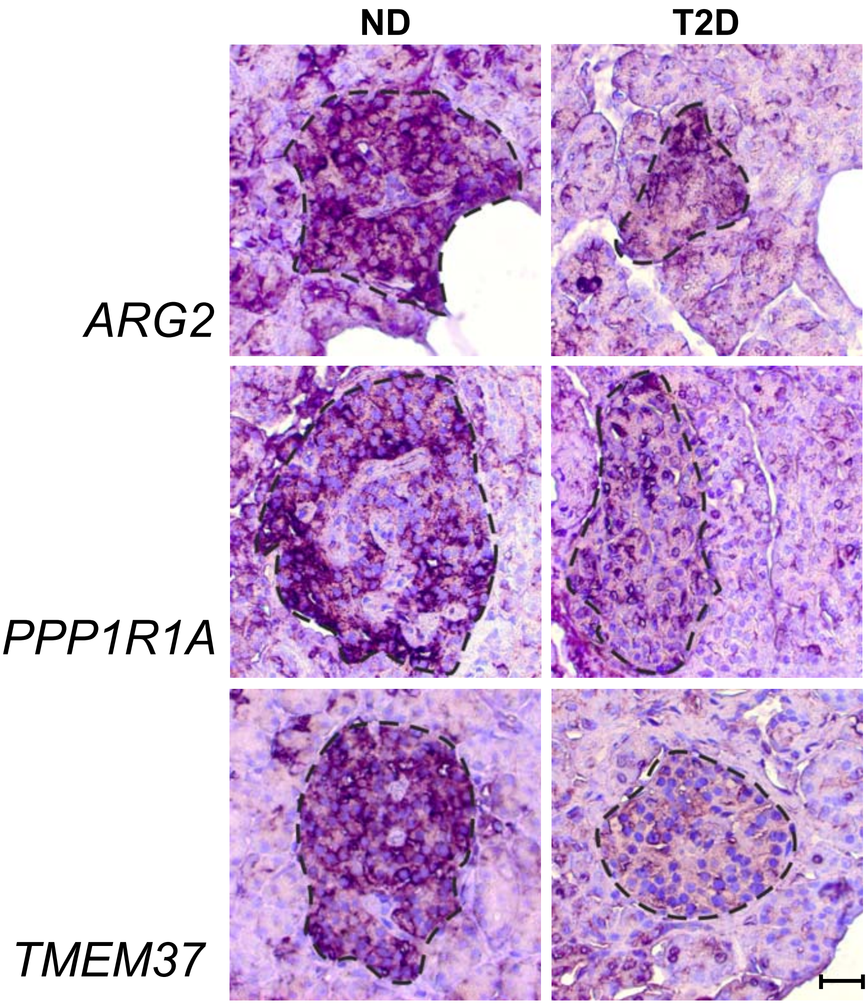

ESM Fig. 3 *In situ* RT-PCR for *ARG2*, *PPP1R1A*, and *TMEM37* expression in ND pancreatic sections. Related to Fig. 3 and Table 2, which report RT-PCR results for the these three transcripts and the full list of differentially expressed genes, respectively. The dashed black lines indicate the margins of the islets. The images are representative of 80 islets analysed in each group. ND, non-diabetic; T2D, type 2 diabetes. Scale bar for all panels: 25µm

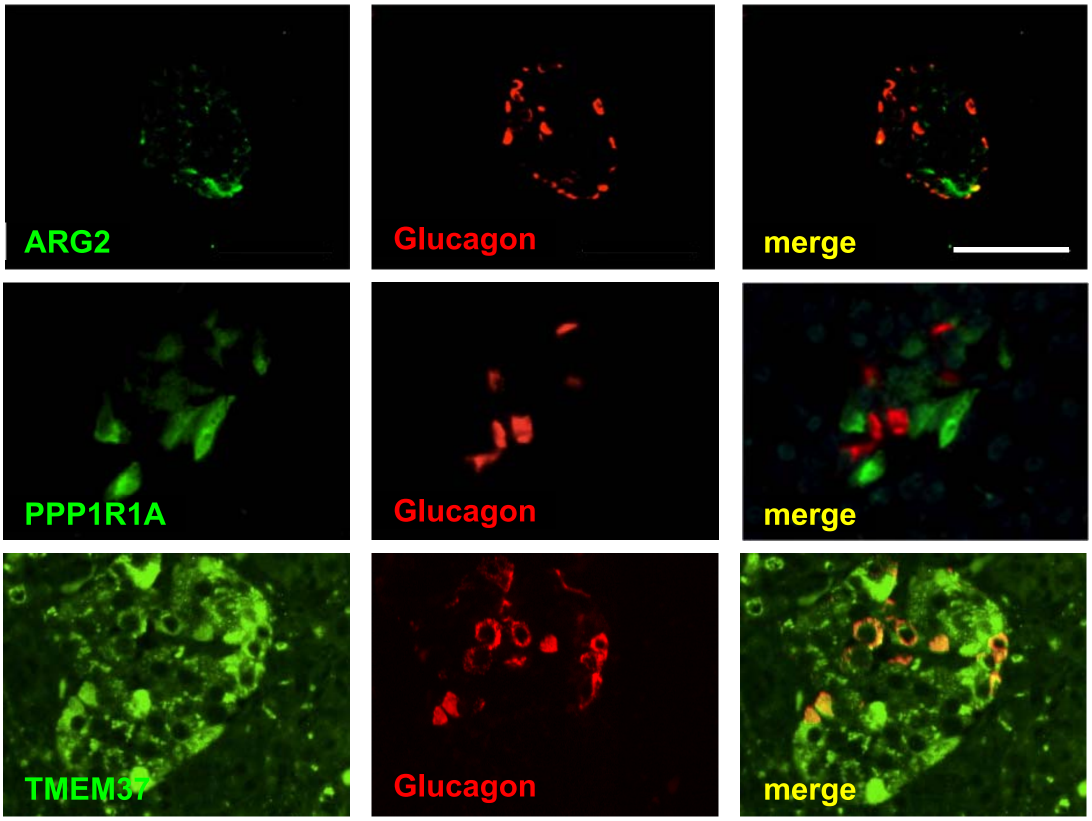

ESM Fig. 4 Confocal microscopy of human pancreas sections for ARG2, PPP1R1A and TMEM37 in addition to glucagon. Related to Fig. 3 and Table 2. The immunostaining was performed on pancreatic tissue that were fixed in paraformaldehyde and embedded in paraffin. Scale bar for all panels: 100µm

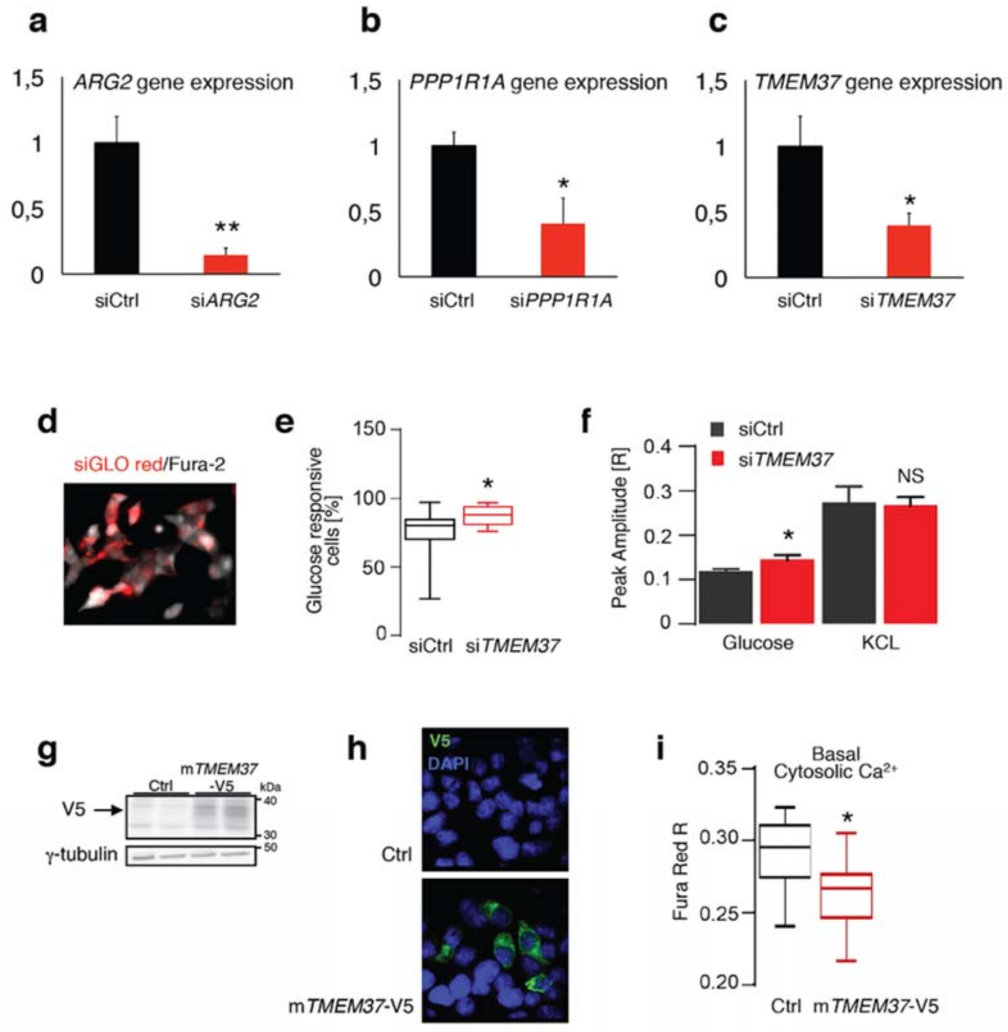

ESM Fig. 5 Functional validation of ARG2, PPP1R1A and TMEM37 in insulin-producing cells. Related to Fig. 3. (a–c). Silencing of *ARG2* (a), *PPP1R1A* (b) and *TMEM37* (c) in INS-1 832/13 cells was confirmed by RT-qPCR. Expression levels were normalized to those of *beta-actin* mRNA. (d). Image of INS-1 832/13 cells transfected with siGLO^{+} (red) and loaded with Fura-2 AM (grey). (e). Boxplot showing the median (line), 25th and 75th percentiles (box), and minimum–maximum (whiskers) percentage of *siTMEM37*- or *siCrtl*-treated INS-1 832/13 cells with changes in cytosolic Ca^{2+} concentrations in response to glucose. (f). Peak Ca^{2+} amplitudes in *siTMEM37*- or siCrtl-treated INS-1 832/13 cells in response to high glucose or high KCl (NS, p>0.05, *p<0.05, **p<0.01, Mann–Whitney U test). (g). Immunoblots for V5 and gamma-tubulin in *TMEM37-V5*-transfected and control INS-1 832/13 cells. (h). Immunostaining for V5 (green) in *TMEM37-V5*-transfected and control INS-1 832/13 cells. Nuclei were counterstained with DAPI (blue). (i). Boxplot showing the median (line), 25th and 75th percentiles (box), and minimum–maximum (whiskers) basal cytosolic Ca^{2+} concentrations in 332 siCrtl-treated (n=10

coverslips) and 419 *siTMEM37*-treated (n=12 coverslips) INS-1 832/13 cells (**p*<0.05, unpaired two-tailed *t*-test).

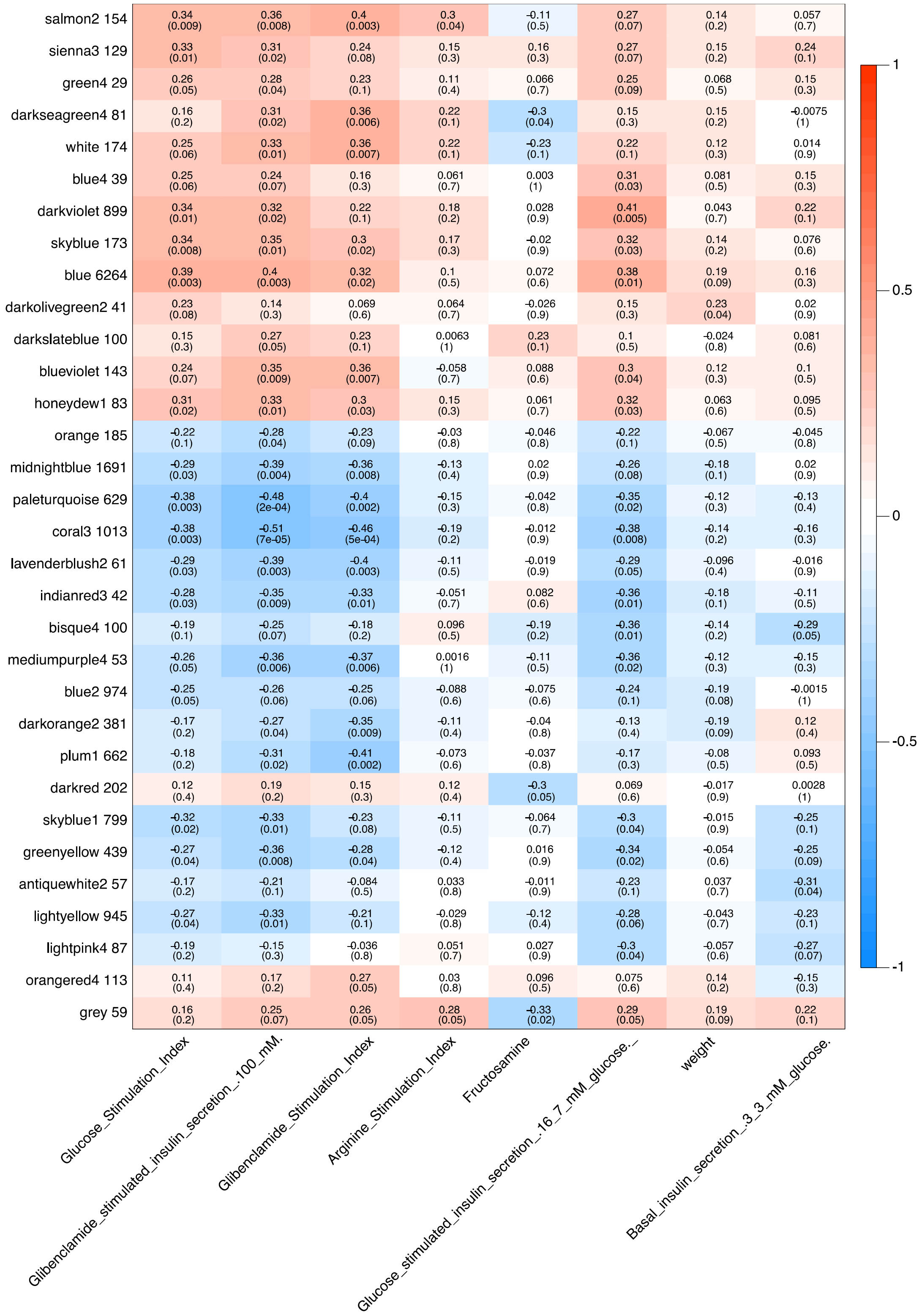

ESM Fig. 6 OD-islet co-expression module correlations with clinical and functional traits. Only module-trait correlations with $p \leq 0.05$ are shown. The colours indicate the strength of the

correlation (blue=strong negative correlation; red=strong positive correlation). Glucose/glibenclamide/arginine stimulation index refers to the glucose/glibenclamide/arginine-induced insulin stimulation index.

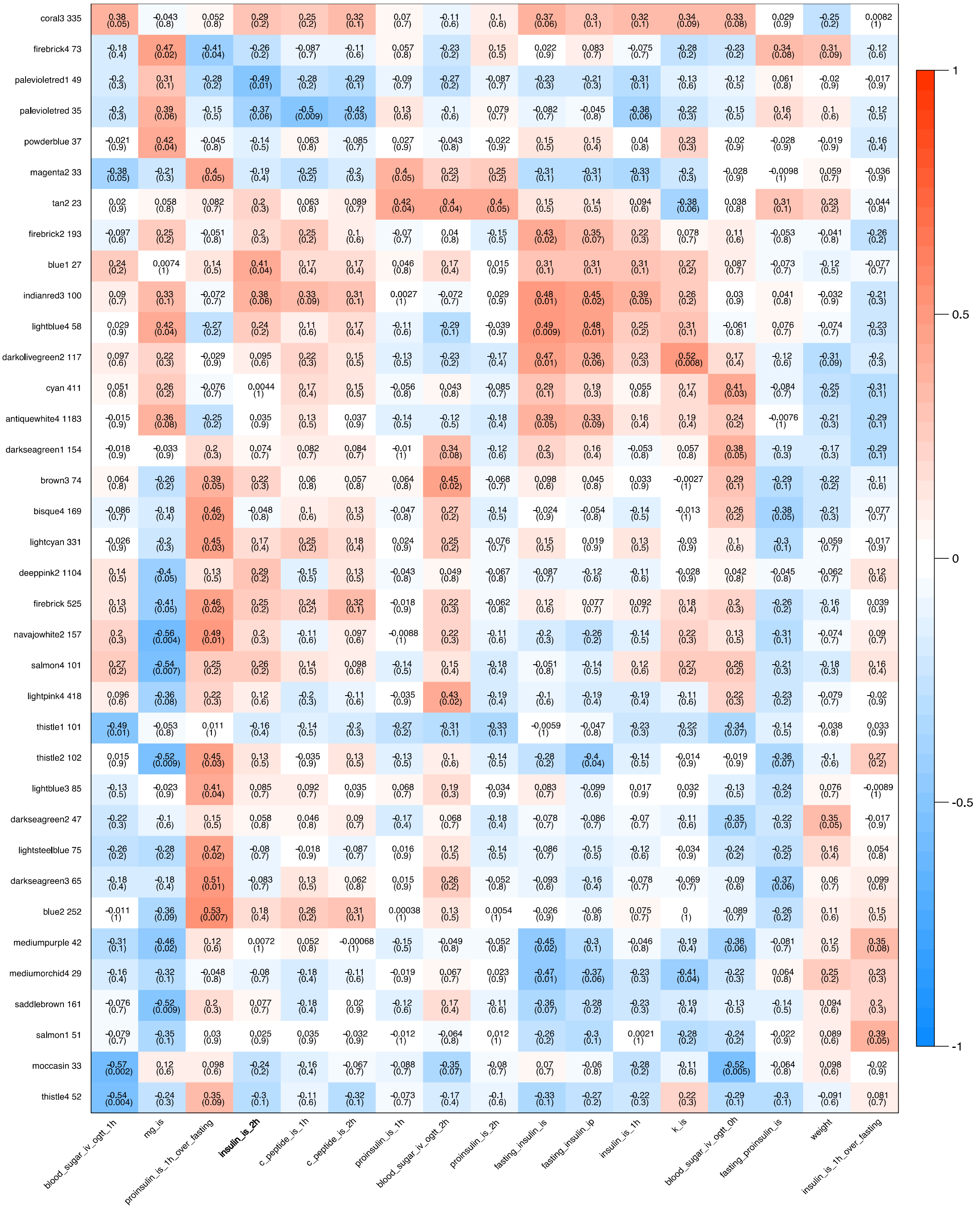

ESM Fig. 7 PPP-islet co-expression module correlations with clinical and functional traits. Only module-trait correlations with $p \leq 0.05$ are shown. The colours indicate the strength of the correlation (blue=strong negative correlation; red=strong positive correlation).

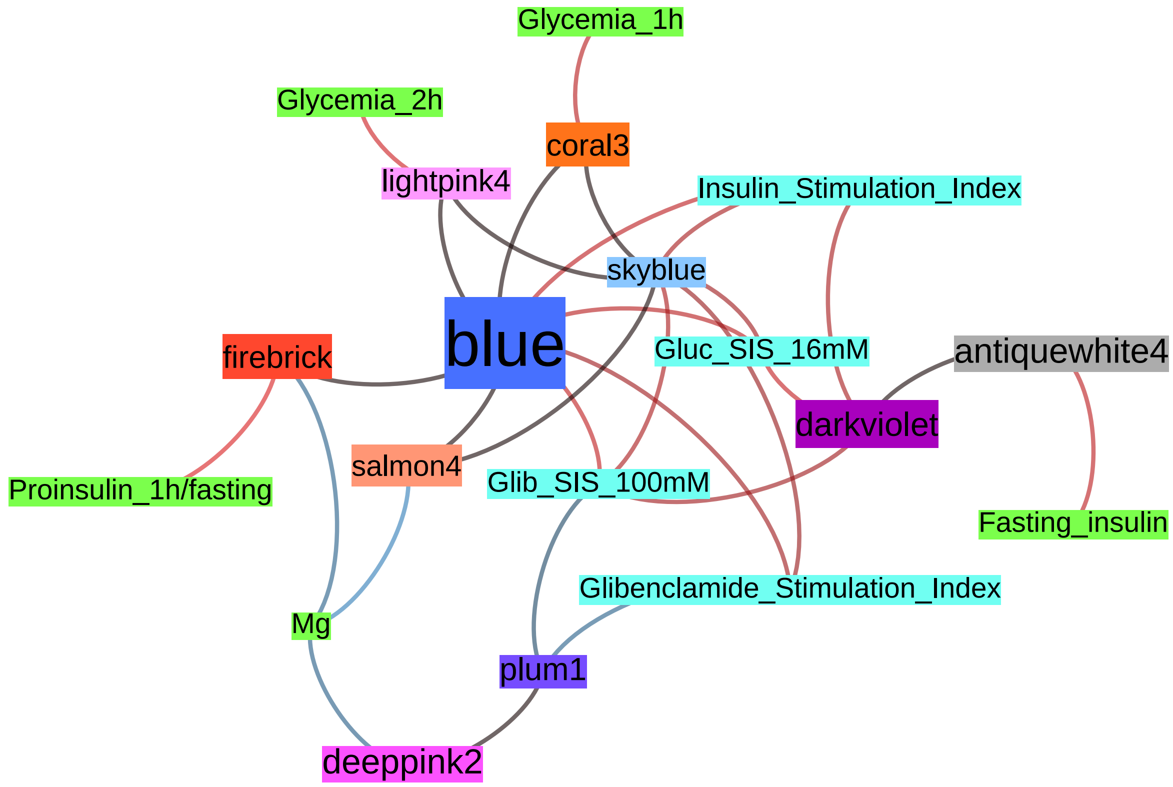

ESM Fig. 8 Module-trait heatmap showing OD and PPP module correlations with functional and clinical traits. The modules (blue, skyblue, darkviolet, antiquewhite4, coral3, lightpink4, firebrick, salmon4, plum1 and deeppink2) are coloured according to their names. OD traits Glucose-induced insulin stimulation index (insulin_stimulation_index), Glucose-induced insulin secretion at 16mM (Gluc_ISI_16mM), Glibenclamide stimulation index and Glibenclamide-induced insulin secretion at 100mM (Glib_ ISI_100mM) are coloured in turquoise and are connected to OD modules (blue, skyblue, darkviolet, plum1). PPP traits Glycemia (OGTT) at 1 or 2 hours (Glycemia_1h/2h), fasting insulin, proinsulin at 1 hour/fasting proinsulin and magnesium (Mg) are coloured in light green and are connected to PPP modules (firebrick, salmon4, lightpink4, coral3, deeppink2, antiquewhite4). Red edges indicate positive correlations between modules and traits; blue edges represent negative correlations between modules and traits. Dark grey edges between modules represent significant overlap between module genes.

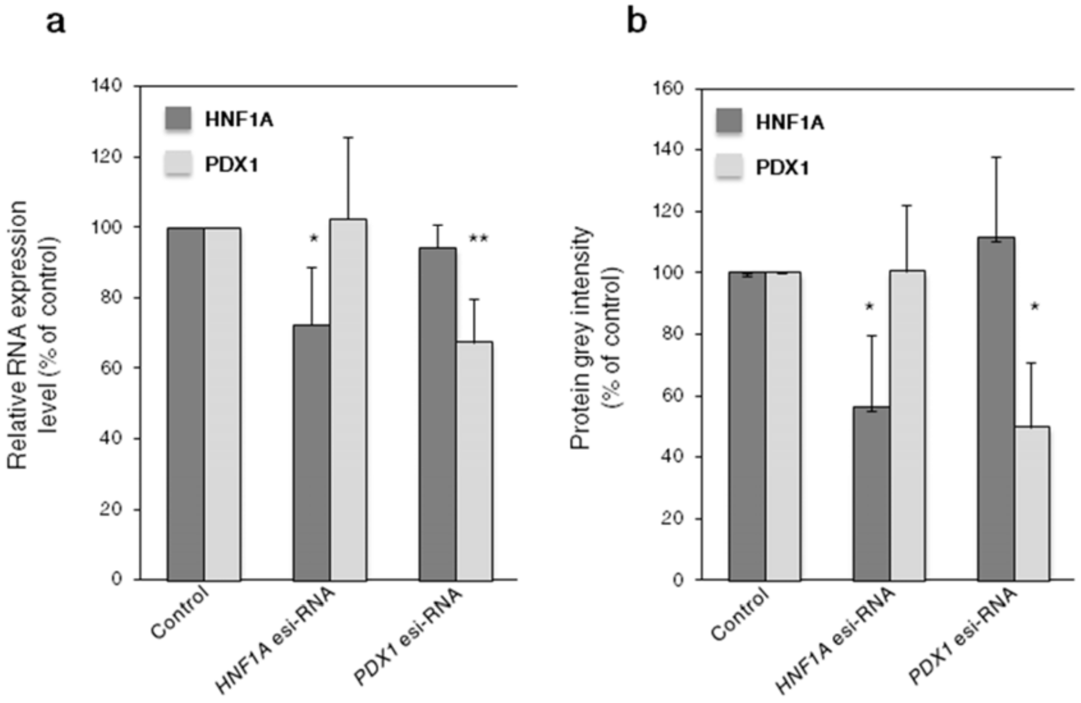

ESM Fig. 9 Efficiency of *PDX1* and *HNF1A* esiRNA-induced silencing in EndoC-βH1 cells. Related to Fig. 6. (a). RT-qPCR. (b). Immunoblotting (Student's *t*-test, *p<0.05, **p<0.01).

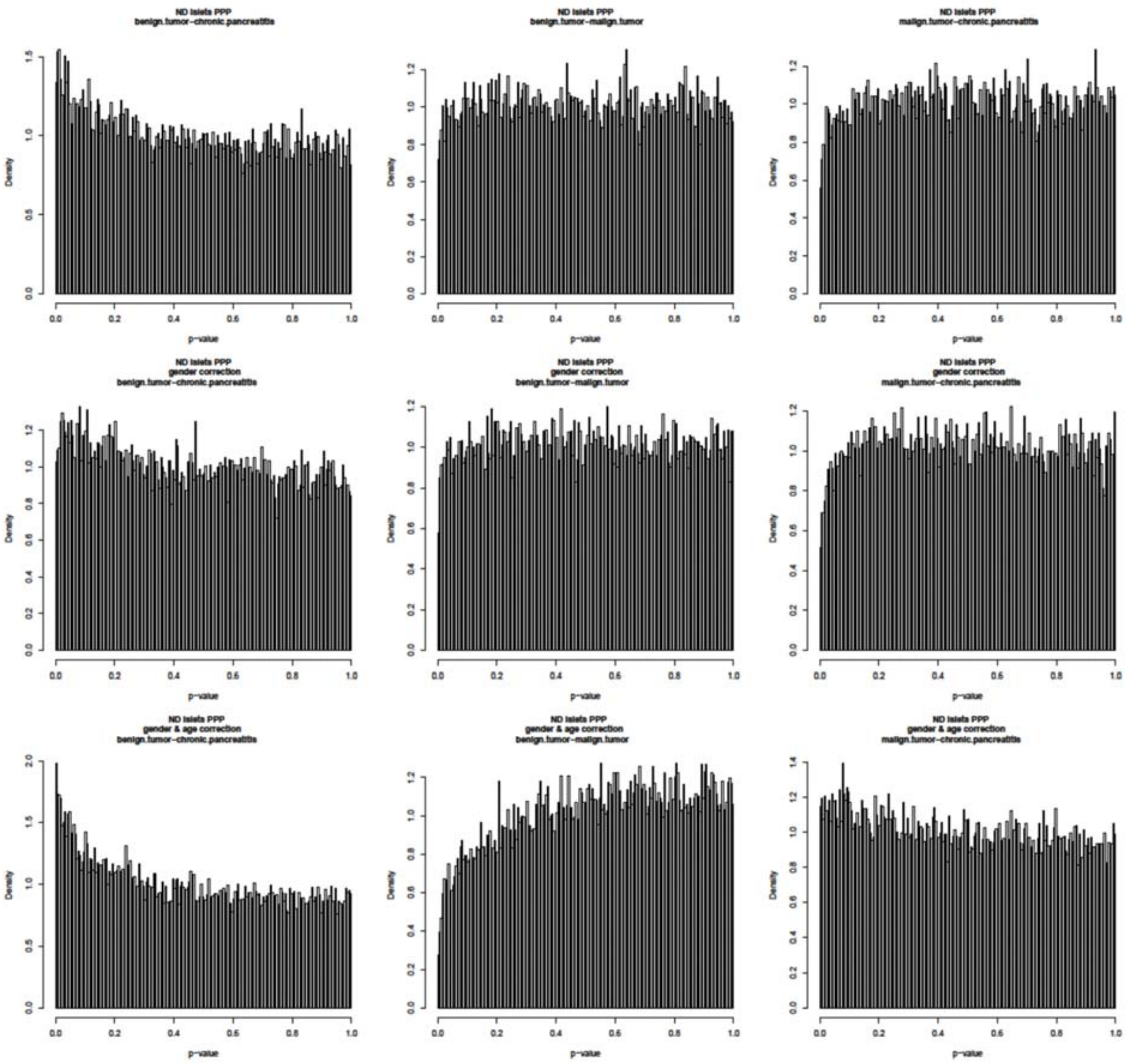

ESM Fig. 10 Linear regression for pancreatic disease in islets from ND PPP. Related to Fig. 2 and Table 1. Islets were obtained by LCM. Linear regression was performed without and with adjustment for age and sex. Most *p*-value distributions follow a nearly uniform distribution, showing only a mild enrichment of low *p*-values. Linear regression for benign tumour versus chronic pancreatitis, with adjustment for age and sex, shows the highest enrichment of low *p*-values (60 probe sets with $p \leq 0.001$ and 0 probe sets with an FDR of ≤ 0.05). ND, non-diabetic.

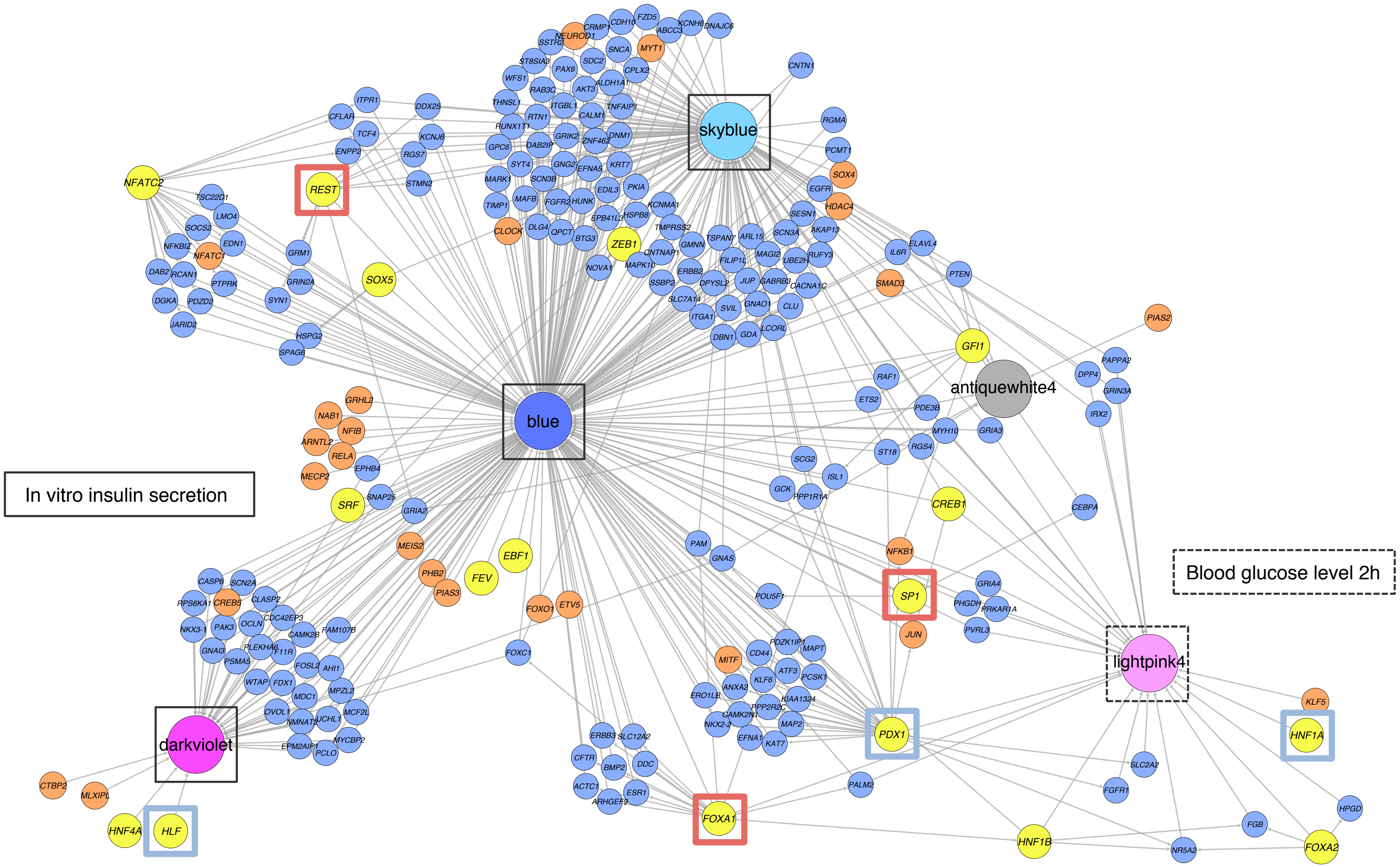

ESM Fig. 11. Detailed view of Fig. 5.

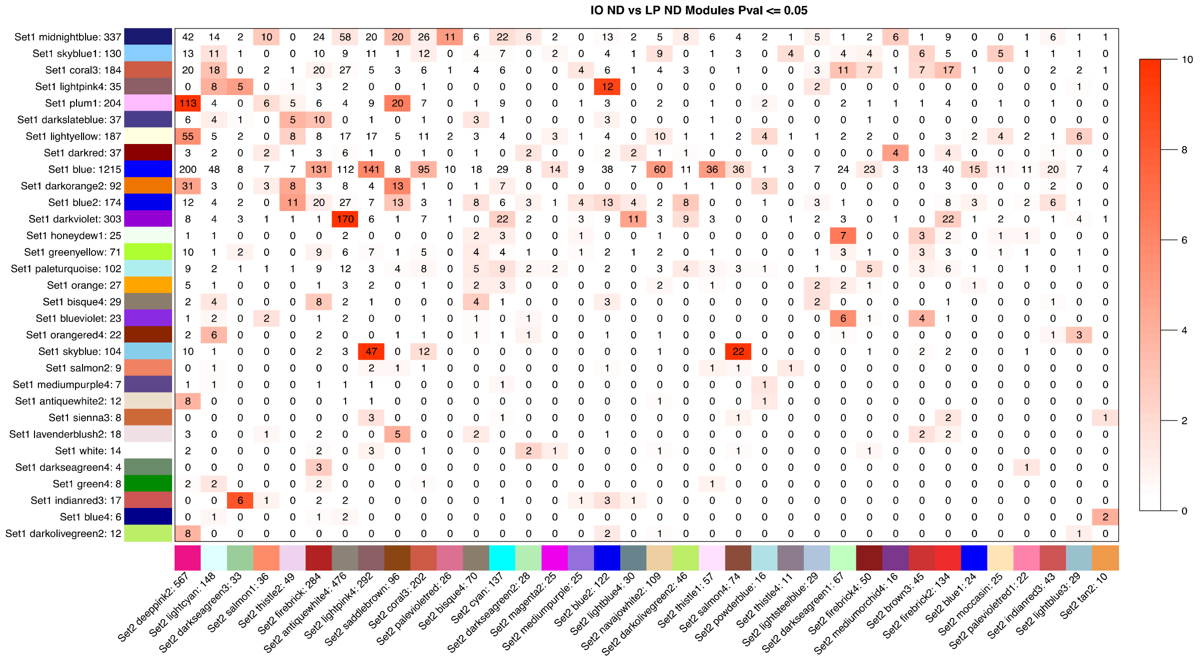

ESM Fig. 12 Comparison of OD-islet modules (Set1) and PPP-islet modules (Set2). Numbers indicate number of overlapping genes. The intensity of red indicates the significance: darker red=more significant (Fisher test). Scale (right) is −log10 (p-value).
